# A cell-based phenotypic library selection and screening approach for the de novo discovery of novel functional chimeric antigen receptors

**DOI:** 10.1038/s41598-022-05058-5

**Published:** 2022-01-21

**Authors:** Julie K. Fierle, Johan Abram-Saliba, Vasileios Atsaves, Matteo Brioschi, Mariastella de Tiani, Patrick Reichenbach, Melita Irving, George Coukos, Steven M. Dunn

**Affiliations:** 1 LAbCore Immunoglobulin Discovery Platform, Department of Oncology, Ludwig Institute for Cancer Research Lausanne, University of Lausanne, 1066 Epalinges, Switzerland; 2 Department of Oncology, Ludwig Institute for Cancer Research Lausanne, University of Lausanne, 1066 Epalinges, Switzerland; 3 Department of Oncology, Ludwig Institute for Cancer Research Lausanne, Lausanne University Hospital and University of Lausanne, 1005 Lausanne, Switzerland; 4 Department of Oncology, Centre Hospitalier Universitaire Vaudois (CHUV), 1011 Lausanne, Switzerland; 5 Department of Oncology, Ludwig Institute for Cancer Research Lausanne, Lausanne University Hospital and University of Lausanne, 1066 Epalinges, Switzerland

**Keywords:** Sarcoma, Immunology, Immunotherapy, Cell therapies, Biologics, Antibody fragment therapy, Drug discovery, Drug screening, Phenotypic screening, Cancer, Cancer therapy, Cancer immunotherapy

## Abstract

Anti-tumor therapies that seek to exploit and redirect the cytotoxic killing and effector potential of autologous or syngeneic T cells have shown extraordinary promise and efficacy in certain clinical settings. Such cells, when engineered to express synthetic chimeric antigen receptors (CARs) acquire novel targeting and activation properties which are governed and orchestrated by, typically, antibody fragments specific for a tumor antigen of interest. However, it is becoming increasingly apparent that not all antibodies are equal in this regard, with a growing appreciation that ‘optimal’ CAR performance requires a consideration of multiple structural and contextual parameters. Thus, antibodies raised by classical approaches and intended for other applications often perform poorly or not at all when repurposed as CARs. With this in mind, we have explored the potential of an in vitro phenotypic CAR library discovery approach that tightly associates antibody-driven bridging of tumor and effector T cells with an informative and functionally relevant CAR activation reporter signal. Critically, we demonstrate the utility of this enrichment methodology for ‘real world’ de novo discovery by isolating several novel anti-mesothelin CAR-active scFv candidates.

## Introduction

The immunotherapeutic concept of actively redirecting the potent effector functions of a patients endogenous or adoptively transferred T cells in order to combat the growth of malignant neoplasms is well established^1–4^. Two classical paradigms that employ distinct molecular strategies have evolved to harness this aspect of natural immunity: soluble dual-specificity proteinaceous drugs, exemplified by bi-specific T cell engagers (BiTE^©^s), or T cells that have been engineered ex vivo to express modified surface receptors (so-called chimeric antigen receptors, or CARs). Both approaches seek to manipulate or reprogramme large populations of T cells such that they can efficiently engage with, and kill, tumors expressing specific disease associated antigens. This is achieved through the physical bridging of both tumor and T cells, leading to the formation of productive immune synapses and the subsequent triggering of T cell-mediated killing and other effector functions^5–8^. To-date, clinical approval has been granted to one BiTE^©^ (blinotumomab) and four CAR therapies (axicabtagene ciloleucel; tisagenlecleucel; brexucabtagene autoleucel; lisocabtagene maraleucel), all for the treatment of CD19^+^ hematological malignancies. A plethora of additional BiTE^©^ and CAR candidates are currently in development targeting several diverse antigens associated with both hematological and solid tumors^9–13^.

The functional specificity and activation signal transduction potential of a CAR is strongly influenced by the choice, distribution, context and cell surface density of the cognate target antigen, and also by those factors that determine optimal cell bridging and synapse formation. These include the affinity and specific epitope recognition properties of the chosen antigen binding domain (typically an scFv), and its organisational presentation, determined by the choice and properties of the T cell membrane-proximal linker/spacer peptide. Thus, for a given CAR scaffold with fixed cytosolic activation/stimulation domains, manipulating one or both of these extracellular aspects can significantly affect functional performance^14–18^. Typically, however, scFvs employed in CAR applications are often reverse-engineered from murine-derived high-affinity IgGs, or obtained via in vitro display technologies with candidate enrichment and selection being largely dominated by affinity considerations. It is not uncommon for such ‘repurposed’ molecules to display unexpected or unfavourable properties when formatted as chimeric antigen receptors, with common issues being poor T cell surface expression/stability and deleterious auto(tonic)-activation^19–22^. Although subsequent tuning of any resulting CAR response can be achieved by empirical evaluation of various spacer and intracellular domains, an over-reliance on the availability of ‘off-the-shelf’ high(er) affinity scFvs may considerably restrict the potential for clinical CAR development and optimization. More recent insights have confirmed that CAR potency and in vivo performance is not strongly correlated with scFv (or ligand) affinity, with low to modest affinity clone variants showing superior performance over high affinity parents in terms of expansion and persistence, and in alleviating on-target/off-tumor toxicities^17,23–25^. Collectively, these observations suggest that many context-sensitive CAR-active clones of interest may fall below the screening thresholds applied during classical in vivo and in vitro antibody discovery cascades, or fail to perform adequately as surface receptors in mammalian cells. Screening paradigms that thus prioritise functional or phenotypic responses may be preferable in this regard. Indeed, several studies have sought to explore the utility of cell-based reporter systems for identifying useful candidate scFv CAR clones. Alonso-Camino *et al.* were initially able to demonstrate that Jurkat cells transfected with a validated murine anti-CEA scFv chimeric immune receptor could be specifically enriched relative to an excess of irrelevant control receptor following stimulation with CEA-engineered HeLa cells and monitoring of the CD69 activation marker^26^. A follow on study used a small Jurkat ‘CARbody’ library to identify a single clone able to trigger IFNγ secretion in the presence of a heterogeneous tumor cell (HeLa) ‘target’^27^. However, the extent of any cross-reactivity to other cell types, killing potency, and the nature of the specific HeLa cell antigen was not reported. Subsequent evolution of this Jurkat phenotypic screening methodology has incorporated technical improvements in vector design; and alternative CAR transfection strategies, expression tags and T cell activation markers^28–30^. Recently, a sophisticated, sequential CRISPR-Cas9 engineering approach was devised in order to ‘tune’ the functional antigen recognition strength of an existing anti-HER2 antibody^23^. The authors were able to confirm a clone enrichment discordance between CAR cell sorting on the basis of soluble antigen binding and that due to phenotypic activation/signalling, and could identify individual VH CDR3 mutations that dramatically reduced apparent scFv affinity while conferring an improved CAR discrimination for a HER2^high^ cell line.

Despite such encouraging studies using model antibodies, it has yet to be demonstrated that phenotypic reporter approaches have practical utility for the de novo discovery of novel, functionally active scFvs CAR leads, and that they can replace or complement classical affinity-driven screening paradigms. Here, we develop a minimal Jurkat-based activation screening methodology and demonstrate its successful application for the isolation of new scFv-CARs targeting the tumor associated antigen mesothelin (MSLN). From large starting naïve library repertoires, we show that phenotypically enriched clones are functionally active by design, recognising and killing cell lines bearing the target antigen at endogenous levels and in native cell surface contexts.

## Results

### Development of a robust covalent CAR-fusion reporter system

**Figure 1:**
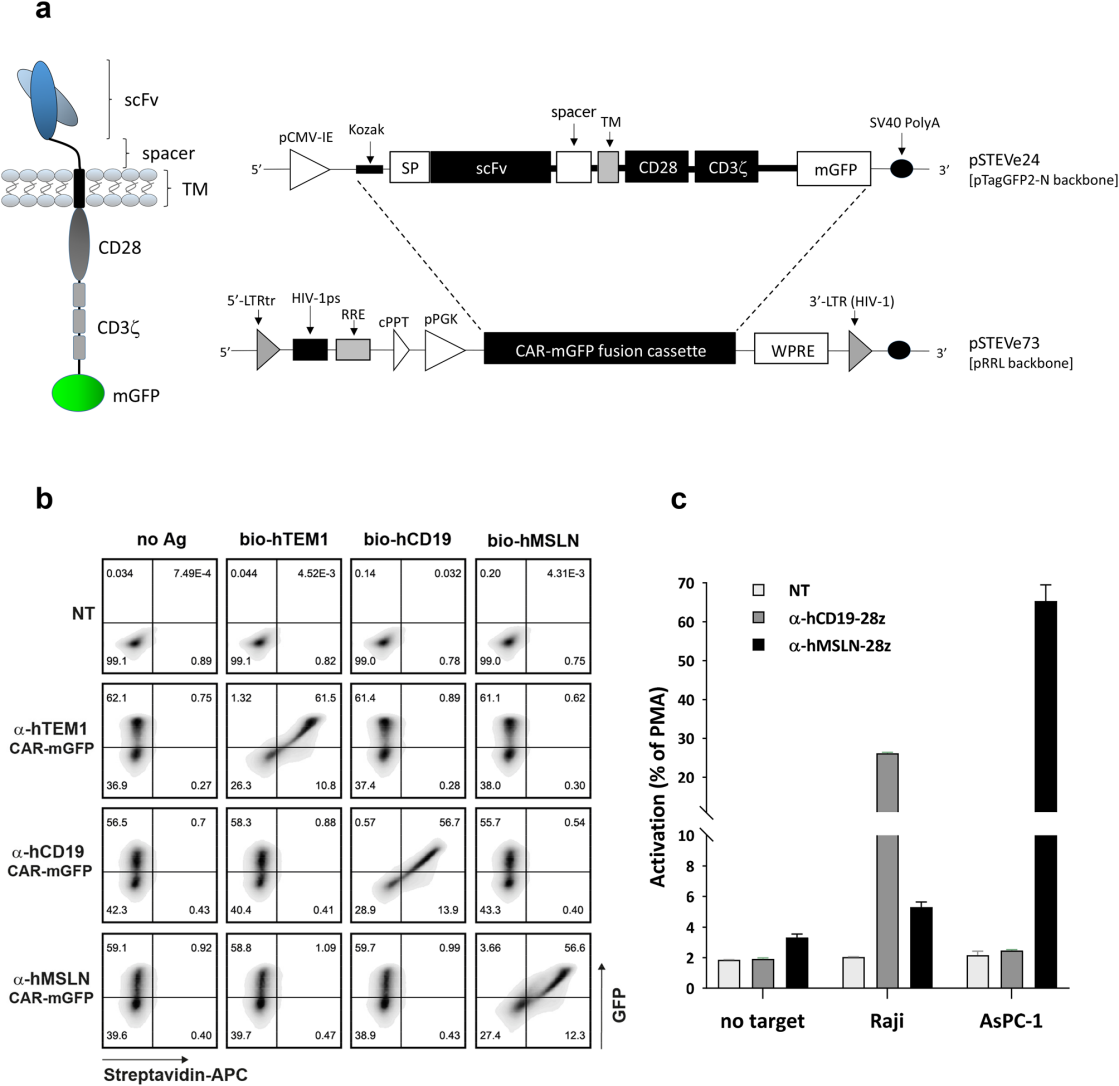
Design and functional validation of a modular CAR-mGFP single-ORF fusion. (**a**) Schematic of the plasmid and lentiviral CAR vector systems used during this study. The designed CAR-mGFP cassette allows for modular cloning of individual or ‘bulk’ scFv populations. (**b**) HEK293-6E cells transiently transfected with various pSTEVe24 CAR-mGFPs selectively bind their cognate antigens as shown by FACS. Binding is seen to correlate with GFP expression. (**c**) Jurkat NFAT-luciferase reporter cells transduced with pSTEVe73 CAR-mGFPs recognising either CD19 (present on Raji) or MSLN (present on AsPC-1) are activated in the presence of target cells expressing endogenous cognate target antigens. PMA, phorbol 12-myristate 13-acetate (100% positive activation control); Data are represented as mean ± SD from triplicate discrete assay points.

Initially, we sought to simplify the recombinant cell reporting system for the assessment of transfection (or transduction) efficiency by FACS. To this end, we explored whether a generic 2nd generation modular CAR scaffold could tolerate a direct translational fusion to a fluorescent protein (FP) reporter as a contiguous multi-domain polypeptide. Whereas 2A self-cleavage and IRES-based systems can lead to the functional and spatial uncoupling of the CAR moiety from the co-expressed reporter epitope (typically, a truncated CD34 or EGFR allowing magnetic cell sorting or FACS staining^28,30^) or intracellular FP, the direct fusion of the CAR to a suitable reporter may be expected to result in a tighter correlation between the two functions. In order to circumvent the known propensity of many FP proteins to dimerize weakly or form higher order oligomers, potentially leading to undesirable aggregative clustering and signalling of CAR fusion molecules in the absence of antigen target, we used a mutated monomeric variant of GFP (mGFP) and fused this downstream of a modular scFv-28ζ 2nd generation CAR cassette separated by a 13-amino acid synthetic cloning spacer. This single-chain ORF was subsequently integrated into both plasmid and lentiviral transduction vectors (Fig. 1a). We cloned three previously described benchmark scFvs recognising different antigens into the pSTEVe24 transfection plasmid cassette under control of a CMV promotor. The resulting scFv-CARs were transiently expressed in suspension HEK293-6E cells and, following staining with soluble biotinylated cognate antigen, we observed a strong correlation between mGFP reporter expression and selective scFv target binding (Fig. 1b). Two of these scFv clones, FMC63 and P4, target human CD19 and human mesothelin (MSLN) ECDs respectively, and have been shown to drive potent CAR activity^31,32^. We therefore virally transduced the corresponding single-ORF CAR-mGFPs into a Jurkat reporter cell line containing luciferase under the control of an inducible NFAT promotor. When challenged with target cells expressing endogenous levels of the cognate antigens, both CARs triggered NFAT signalling in a target cell selective manner (Fig. 1c). This data thus demonstrates that a generic CAR architecture can tolerate a covalently appended intracellular FP and that spatial separation/segregation of the transduction reporter component is not mandatory to ensure the presentation and structural integrity of the targeting scFv, or the intracellular functioning of the CD3ζ-mediated CAR signalling apparatus.

### Functional CAR-mGFP fusions can be enriched by phenotypic activation and FACS sorting

**Figure 2:**
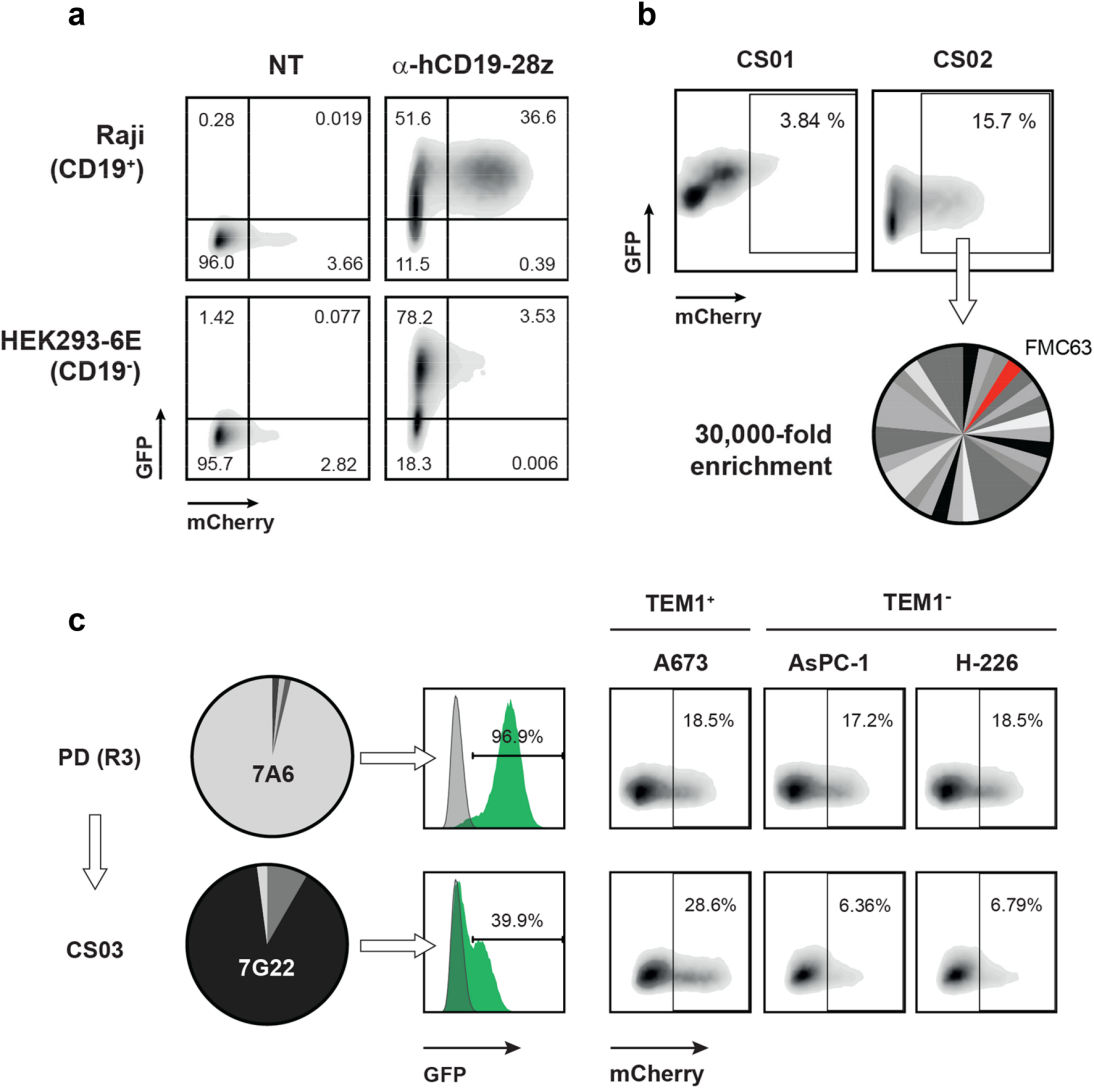
Phenotypic enrichment of functional CAR-mGFPs in model systems. (**a**) Jurkat NFAT-mCherry reporter cells containing an anti-CD19 CAR-mGFP are selectively activated by co-incubation with CD19^+^ Raji cells as shown by FACS. (**b**) Anti-CD19 CAR-mGFP Jurkats spiked at 1:10^6^ into a complex irrelevant CAR-mGFP background are strongly enriched following two rounds of phenotypic cell sorting (CS). (**c**) Phenotypic cell sorting rescue of a novel and selective CAR-active anti-TEM1 scFv from a heavily enriched phage display (PD) output. Pies indicate relative frequency of clone 7A6 in the transduced Jurkat starting library and of 7G22 following three rounds of phenotypic CS. Transduction efficiencies of these two CAR-mGFP clones into Jurkat NFAT mCherry cells was determined by FACS gating on GFP-positive cells, with activation in the presence of TEM1-expressing A673 target and non-target control cells reported by mCherry induction.

We next sought to evaluate a Jurkat cell line which had been engineered to contain a T cell activation-inducible mCherry FP under the control of a tandem NFAT promotor, which potentially would permit the FACS-based sorting of CAR-containing cells presenting a minimal activation phenotype. To explore the potential of this system to phenotypically enrich CAR-active clones of interest we first transduced Jurkat NFAT-mCherry with FMC63 CAR-mGFP and confirmed by FACS that CD19^+^ Raji cells could stimulate the GFP^+^ Jurkat population to express mCherry (Fig. 2a). We next spiked these FMC63 CAR cells at a ratio of 1 copy per million into a population of NFAT-mCherry Jurkats that had been virally transduced with an irrelevant and diverse CAR-mGFP repertoire originating from a large naïve phage display (PD) scFv library that had been panned for two rounds (R2) against three unrelated recombinant target antigens. In order to minimize the occurrence of multiple lentiviral co-transduction events with different clones, MOI titrations were conducted to ensure a transduction frequency of ~ 20%. GFP^+^ Jurkat cells were harvested by FACS sorting, expanded, and challenged against CD19-negative HEK293-6E cells in a negative sorting step to deplete clones triggering non-specific mCherry expression. The expanded ‘clean’ population was then subjected to two positive cell sorting cycles (CS01 and CS02) against CD19^+^ Raji cells, with the mCherry^+^ stimulated population being allowed to recover and expand between cycles. As shown in Fig. 2b, mCherry^+^ cells were significantly enriched following CS02. Bulk recovery of scFv sequences from the harvested CS02 mCherry^+^ cell population was performed using RT-PCR and the proportional occurrence of FMC63, determined by Sanger sequencing of 48 randomly-selected sub-clones, was found to be ~ 3%. Hence, even allowing for the potentially poorer propagation fitness of the murine-derived FMC63 in the presence of a complex fully-human scFv-CAR background, two cycles of phenotypic activation sorting achieved a substantial 30,000-fold enrichment of this CAR clone over the starting spike.

Encouraged by our findings, we asked whether such an approach could rescue low-frequency CAR-active clones present in a de novo phage display selection output enriched against a challenging cell surface antigen. Previously, we had conducted a PD campaign against the membrane proximal sialo-mucin stalk region of human TEM1 (CD248; AAs 547-683) using large human naïve scFv libraries. This fragment possesses a high degree of post-translational *O*-glycosylation and has no predicted structural domain fold. Following two PD selection rounds against a recombinant mucin stalk fusion protein (SpyC-TEM1ΔN) and a third against whole TEM1^+^ cells, we observed that the resulting R3 PD output sequences had converged strongly to just four abundant scFvs with one clone accounting for over 90% of the population. When this dominant clone (7A6) was reformatted as a discrete CAR-mGFP in mCherry reporter cells, we observed only modest NFAT activation in the presence of the cognate A673 TEM1^+^ target cell line, despite high levels of CAR transduction. Moreover, this activation appeared non-selective as comparable activation levels were also observed in the presence of the two TEM1-negative control lines (Fig. 2c). We next PCR-amplified and batch-cloned the scFv population from the entire SpyC-TEM1ΔN R3 PD output into the pSTEVe73 CAR-mGFP cassette prior to generating a lentivirus library and transducing into Jurkat NFAT-mCherry cells. Pre-processing of the resultant cell library was carried out by co-incubation with TEM1-negative control cells (HEK293-6E), followed by FACS to enrich for the non-activated GFP^+^ population. Subsequently, we conducted three sequential cycles of positive activation cell sorting (CS01-CS03) using cognate TEM1^+^ A673 cells as the stimulator line. Batch retrieval of enriched sequences following CS03 revealed that a single clone (7G22), originally present at a low frequency (0.9%) in the residual background of the 7A6-enriched R3 PD starting pool, now accounted for over 80% of all recovered sequences. Reformatting of this clone as a CAR-mGFP confirmed that, in contrast to 7A6, it possessed significant recognition and activation selectivity for TEM1^+^ cells and, moreover, could drive the specific CAR-mediated killing of target cells expressing endogenous levels of cognate antigen (Fig. 2c and Supplementary Fig. S1). Hence, phenotypic activation screening resulted in the dramatic rescue and enrichment of a genuine CAR-active clone at the expense of a seemingly dominant scFv TEM1 binder that was strongly favoured by classical affinity-driven phage display.

### Phenotypic activation sorting identifies novel CAR-active anti-MSLN scFv domains from a minimally selected naïve scFv library

**Figure 3:**
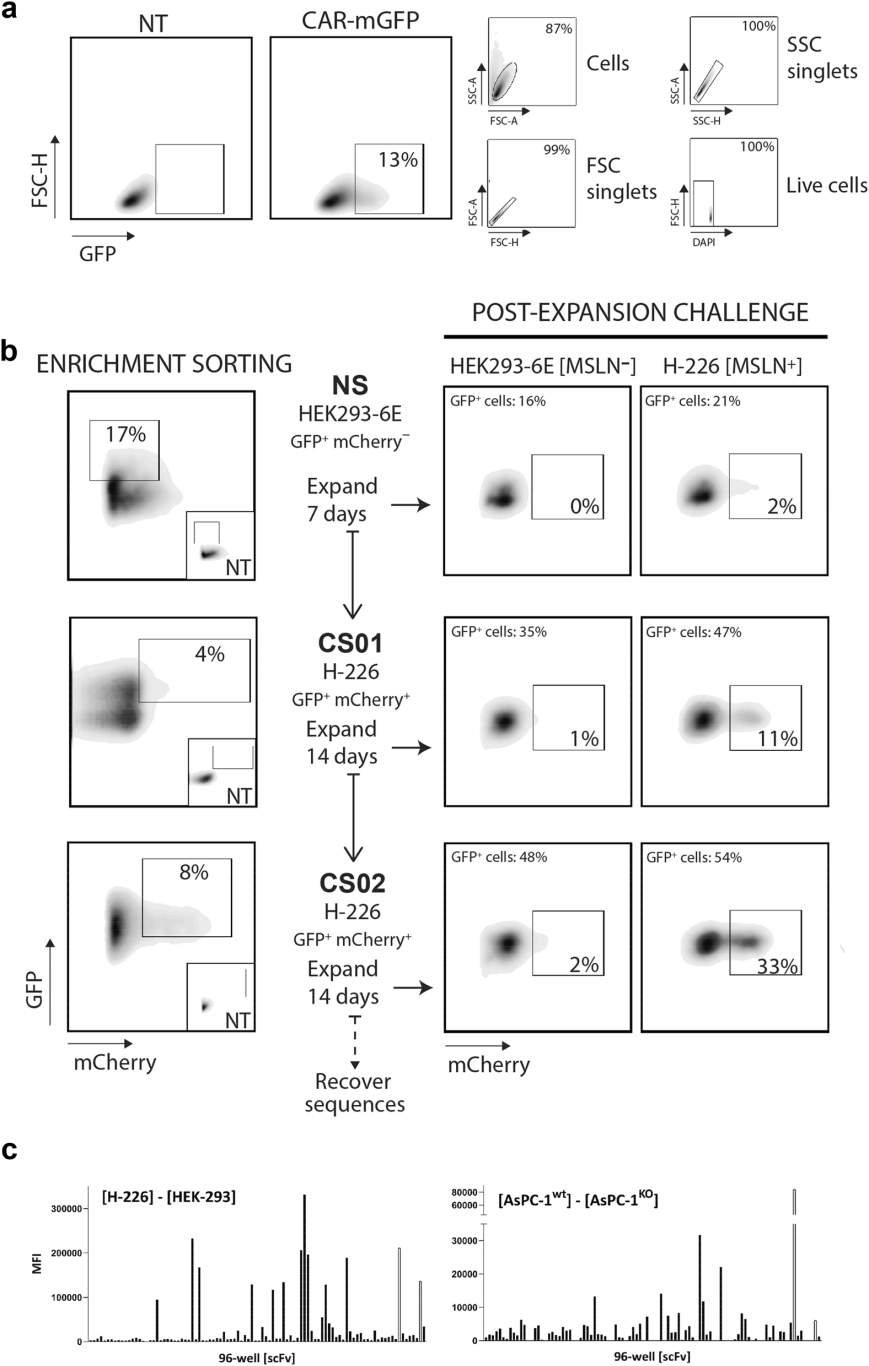
Enrichment of an anti-hMSLN CAR-active clone population by phenotypic sorting. (**a**) Low-frequency transduction of Jurkat NFAT-mCherry cells with a CAR-mGFP library incorporating ~ 10^5^ scFvs pre-selected on MSLN (see text). (**b**) Phenotypic sorting and enrichment workflow for anti-MSLN CAR-mGFP. NS, negative sorting of the transduced library following co-incubation with MSLN^−^ HEK293 cells to subtract for non-MSLN reactive clones (GFP^+^ mCherry^−^ gate); CS01/CS02, positive cell sorting steps to enrich for the Jurkat CAR-mGFP clone population responding to MSLN^+^ target cells (GFP^+^ mCherry^+^ gate). (**c**) Representative iQue flow cytometer plate screening data for MSLN-dependent target cell binding by discrete scFvs recovered from the phenotypic library sorting and expressed to bacterial media. Left panel, Mean Fluorescence Intensities (MFI) for scFvs binding cognate MSLN^+^ H-226 cells with subtraction of the corresponding binding to HEK293 MSLN^−^ control cells; Right panel, Mean Fluorescence Intensities (MFI) for scFvs binding cognate MSLN^+^ AsPC-1 cells with subtraction of the corresponding signals obtained against an AsPC-1 polyclonal line enriched for MSLN KO events by CRISPR. Cell binding of scFvs was detected using anti-Histag-Alexa647. Empty bars, anti-MSLN positive controls.

We next asked whether the same methodology was sufficiently robust to permit the phenotypic isolation of genuine CAR-active scFv clones during a de novo discovery campaign. To this end, we chose to target mature human mesothelin (hMSLN, AA residues 296-606) as a therapeutically relevant cell surface antigen highly expressed in several aggressive solid tumours including mesothelioma and pancreatic adenocarcinomas^33–35^. Starting from a large fully human naïve scFv phage display discovery library comprising some 2 × 10^10^ IgM/IgD-derived members, it was first necessary to collapse the clone population to a size suitable for the practical construction of a transduced Jurkat cell library. We thus performed an initial low stringency phage display R1 ‘capture’ step on bead-immobilized recombinant hMSLN which yielded an eluted ‘binder’ pool of ~ 10^5^ scFvs which were then sub-cloned *en masse* to generate a pooled library of lentiviral CAR-mGFPs. Following viral packaging, Jurkat NFAT-mCherry cells (2 × 10^6^) were transduced at low MOI to yield 13% GFP^+^ cells (Fig. 3a), sufficient to encompass the bulk of the diversity contained within the cloned input library whilst reducing the likelihood of multiple co-transduction events. Subsequently, cells were subjected to a negative sort (NS), concomitantly enriching for both GFP expression and depletion of clones that express mCherry when co-incubated with hMSLN-negative control cells (HEK293-6E). Encouragingly, when this cleaned and expanded GFP^+^ mCherry-negative library population was stimulated with H-226 target cells, a human mesothelioma cell line expressing high levels of MSLN, selective NFAT activation was already evident for ~ 1% of the cells. This CAR-active population was seen to enrich substantially following two cycles of positive cell sorting and expansion against H-226 cells, with the GFP^+^ mCherry^+^ gate representing 33% of the expanded cells at CS02 (Fig. 3b). Bulk sequences were recovered from this CS02 population by RT-PCR and individual scFvs were resolved, expressed in bacterial supernatants and screened for selective recognition of both H-226 and AsPC-1 pancreatic tumor cells by iQue FACS binding assay (Fig. 3c and Supplementary Fig. S2).

**Figure 4:**
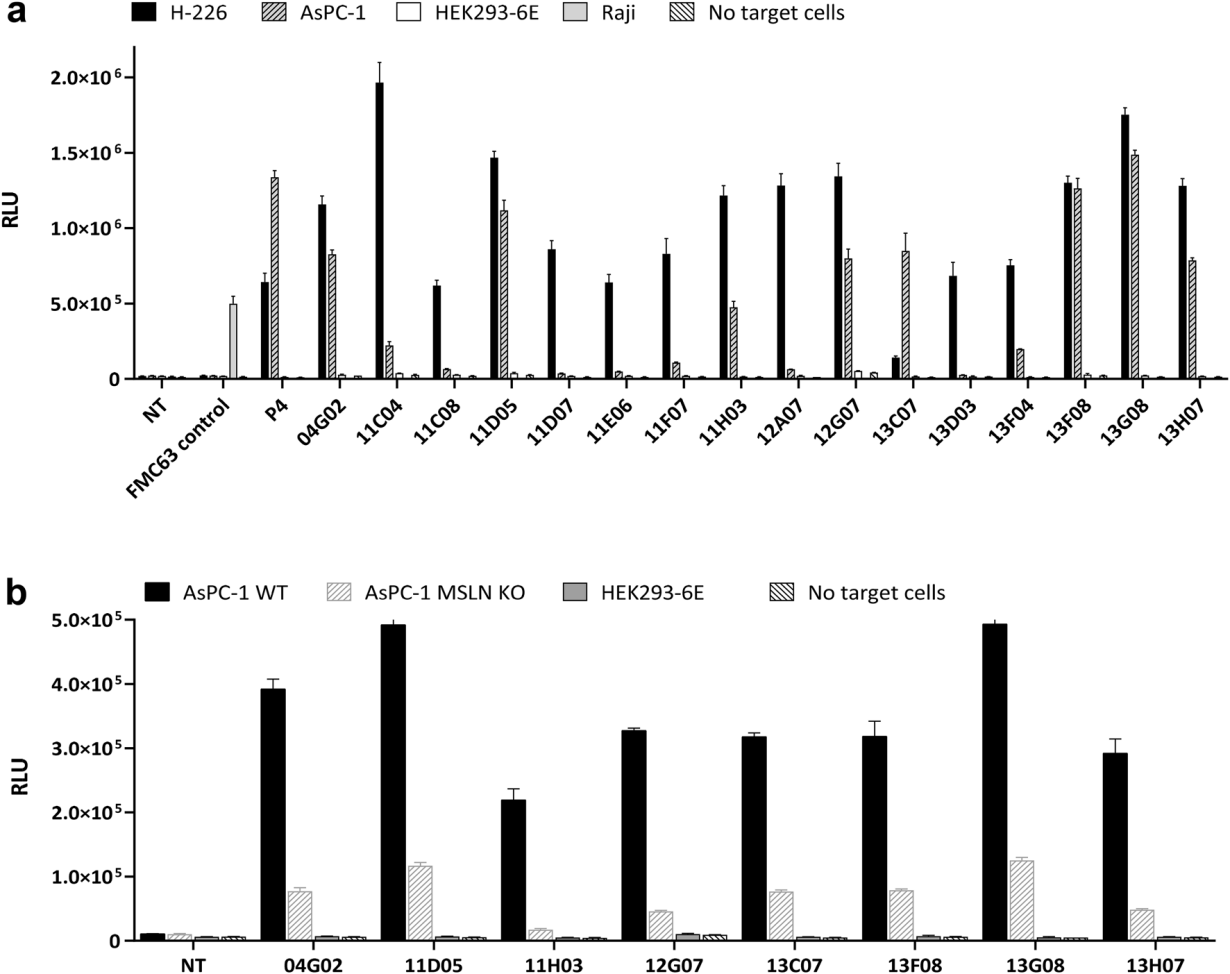
Phenotypically selected clones show specific and differential activation of NFAT signalling in the presence of hMSLN^+^ cells. CAR activation profiling using phenotypically selected scFv clones reformatted as CAR-mGFPs in Jurkat NFAT-Lucia luciferase reporter cells. (**a**) Stimulation in response to MSLN^+^ (H-226, AsPC-1) and MSLN^-^ (HEK293-6E, Raji) target cells. (**b**) Stimulation in response to AsPC-1 wt and a polyclonal AsPC-1 KO cell pool enriched for MSLN CRISPR disruption events. Assay controls: FMC63 (CD19^+^, Raji); P4 (MSLN^+^ lines). RLU, relative light units; data are represented as mean ± SD from triplicate discrete assay points.

Interestingly, despite possessing significantly higher levels of MSLN mRNA, AsPC-1 cells do not typically stain as well as H-226 with anti-MSLN scFvs (Supplementary Fig. S3). Comparative Western blot analysis indeed confirmed that detectable MSLN protein levels were lower in AsPC-1 cell lysates than in H-226, with relative molecular weights indicating clearly distinct pre- and/or post-translational processing of MSLN between the two cell lines. Further, we observed by sandwich ELISA substantially higher levels of MSLN shedding from AsPC-1 cells than from H-226 (Supplementary Fig. S3). Taken together, our observations are consistent with previous studies suggesting that cell context may markedly determine the extent to which certain MSLN surface epitopes are present, accessible, or masked (by, for example, co-expressed Muc16/CA125)^36,37^. Subsequently, recovered scFv clones staining H-226 and/or AsPC-1, including those giving only marginal shifts by iQue assay, were reformatted as a non-redundant panel of CAR-mGFPs and transduced into Jurkat NFAT-Luciferase reporter cells. Co-incubation with both MSLN target and non-target cells revealed a differential and diverse pattern of ‘signal 1’ NFAT activation which was not obviously predicted by the magnitude of soluble recombinant hMSLN ligand binding, transduction efficiency, or raw monovalent affinity for immobilized hMSLN (Fig. 4a, Supplementary Fig. S4 and Supplementary Table S1). Indeed, several CAR-mGFP clones showing negligible or weak staining with soluble hMSLN antigen nevertheless responded appreciably and specifically to the presence of hMSLN^+^ target cells. Additionally, of those clones that could be activated by endogenous wt AsPC-1 cells, all were shown to have substantially attenuated responses in the presence of a CRISPR-engineered AsPC-1 polyclonal cell pool enriched for MSLN gene knock-out events (Fig. 4b). To probe further the recognition characteristics of these various clones, we expressed each of the three predicted ECD domains of mature hMSLN (d1, d2, d3) in isolation as SpyCatcher fusions and profiled their binding using a bead-based multiplexed iQue assay. In common with the P4 reference scFv, several of the activating scFvs from the collated panel specifically recognised the membrane-distal domain 1, although it is apparent that clones targeting domains 2 and 3 were also being selected during the phenotypic sort (Supplementary Fig. S5). Interestingly, clone 13C07, which only drives appreciable NFAT activation in the presence of MSLN presented on AsPC-1, is the sole domain 3 binder in our panel and shows low nM affinity to immobilized MSLN (Supplementary Table S1). As MSLN protein levels on AsPC-1 are lower than for H-226, this would suggest that the apparent differential selectivity of 13C07 is driven by qualitative aspects of MSLN expression, rather than antigen abundance. Additionally, other clones appear to bind specifically to the full length hMSLN ECD but do not recognise the discrete domains, implying that they target conformational or inter-domain epitopes (eg. 11C04). Further, as a membrane anchored CAR, clone 13F08 is triggered by endogenous cell surface MSLN and can be stained with soluble recombinant MSLN. In contrast, however, when expressed as a soluble monovalent scFv, although able to strongly and specifically stain hMSLN^+^ cells (data not shown), this clone did not bind appreciably to bead immobilized recombinant MSLN or its fragments (Supplementary Fig. S5). Such context-dependent recognition of MSLN by antibody fragments has been reported previously^38^.

**Figure 5:**
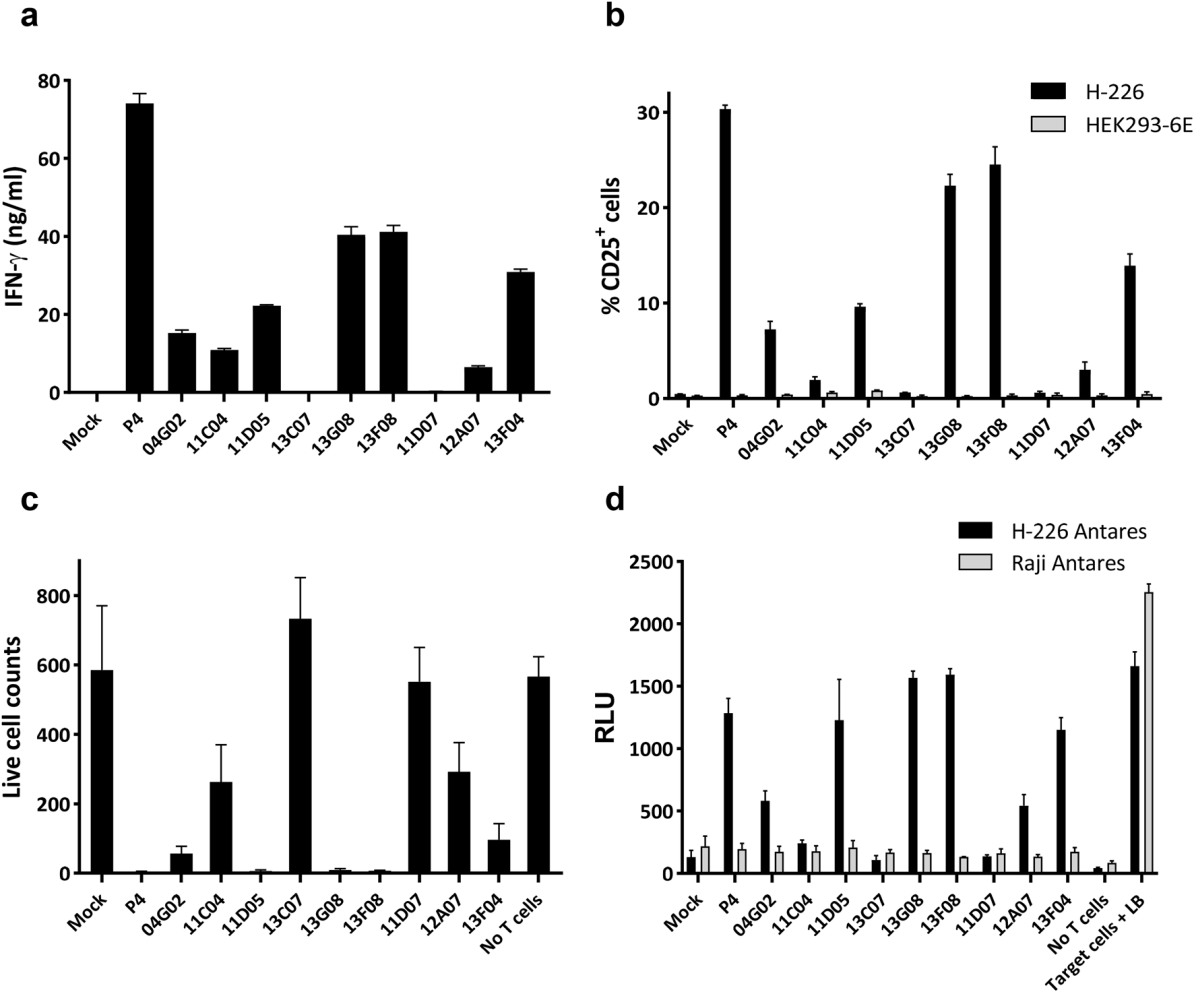
Phenotypically enriched anti-hMSLN CAR-mGFP clones trigger effector and killing functionality in transduced primary T cells. (**a**, **b**) Expression of inflammatory (IFNγ) and a surface activation marker (CD25) in the presence of H-226 tumor cells. (**c**, **d**) Killing of H-226 Antares transduced target cells as determined by gated cell survival (**c**) and liberation of the Nanoluc luciferase reporter by cell lysis (**d**). Mock, out-of-frame CAR-mGFP ORF vector; HEK293-6E and Raji Antares, MSLN^−^ control lines. All data are represented as mean ± SD from triplicate discrete assay points; RLU, relative light units; LB, lysis buffer (100% cell death control). Live Cell Counts in (**c**) indicate remaining DAPI^−^ Antares^+^ target cells.

Encouraged by these observations, we transduced activated primary human pan-T cells isolated from healthy donor PBMCs (Supplementary Fig. S6) with individual CAR-mGFPs and assessed their ability to kill target cells. Several clones, including 13F08, showed clear and specific activation (IFNγ, CD25) in the presence of H-226 cells and demonstrated potent killing as revealed by target cell depletion (determined by iQue), luciferase release from reporter-engineered H-226, and the presence of microscopically observable co-culture killing foci (Fig. 5 and Supplementary Fig. S6). Taken together, this data confirms that phenotypic NFAT-activation library sorting represents a powerful approach for the isolation of novel, functional, and diverse CAR warhead panels, driving the identification of context-selective binders that recognize native antigens in the absence of classical affinity-based enrichment.

## Discussion

The functional efficacy of a synthetic CAR scaffold is determined in large part by the specific characteristics of the extracellular binding moiety, typically an scFv or proteinaceous ligand domain. An accumulating body of evidence suggests that classical antibody isolation paradigms, which are typically configured to result in end-point molecules with high affinity and specificity towards the target epitope, may not yield optimally performing CARs in the in vivo setting, and may not be good starting points for de novo CAR discovery programmes^17,24,25,39^. Indeed, systematic studies investigating the relationship between binding affinity and productive T cell activation indicate that the use of higher affinity CAR ‘warheads’ may carry significant disadvantages by decreasing the ability of engineered T cells to discriminate between antigen levels present in tumors versus healthy cells/tissues^40^. Such high-affinity engagement, typically characterised by slow scFv off-rates, might exacerbate so-called ‘on-target off-tumor’ toxicities and cytokine release syndrome (CRS), and also result in impaired T cell expansion and persistence. Attempts to address these deficiencies through directed mutagenesis have shown promise, however the target epitope of the affinity-attenuated variant CARs remains that of the high-affinity parent and may not be optimal for the bridging of effector cells^17,23,41^.

In order to uncouple raw scFv affinity from antigen recognition whilst simultaneously relaxing specific epitope recognition constraints, we have explored functional phenotypic library screening using scFv clone populations derived from a large fully human naïve phage display library constructed using only the naive IgD/M component of healthy donor repertoires. Such libraries lack somatic VH mutations and typical phage display output clone populations are characterized by average monovalent KDs in the mid-low nM range. By exploiting a minimal, yet physiologically relevant ‘signal 1’ NFAT cell reporter system combined with appropriate negative selection and gating strategies, we show that large scFv repertoires can be directly mined for novel scFv molecules that meet the dual criteria of engaging CAR-compatible target epitopes while possessing appropriate kinetic affinity characteristics to overcome signalling thresholds. This simple read-out efficiently enriches for molecules showing demonstrable efficacy in primary CAR T cell effector assays.

For this validation study, we opted to challenge the CAR-Jurkat libraries with tumor cell lines expressing endogenous levels of native target antigen rather than using engineered over-expressing or KO cells. This, however, requires that negative and positive sorting are conducted on different cell membrane backgrounds which increases the potential risk that clones recognising surface determinants other than the antigen of interest may escape effective negative selection, triggering non-specific Jurkat activation, and masking the enrichment of CAR clones responsive for the antigen of interest. We surmise that the frequency of genuine antigen-specific clones captured in the initial PD R1 library reduction step may be critical in this regard, and that this in turn will be influenced by the nature and quality of the recombinant antigen, the stringency of washing and elution, and the representation of actual binders in the starting phage display library. Hence, we envisage that in some circumstances, the targeting of certain antigen targets or target classes may require a more defined approach with appropriately engineered cells and/or more sophisticated sorting regimes than we present here.

A possible limitation of using an FP such as mCherry in our NFAT reporter system relates to its rapid maturation rate (on-switch) and long half-life (off-switch) once expressed^42^. The rapid inductive accumulation and stable persistence of this FP during the 24 h CAR activation period essentially reflects a yes/no threshold response distribution, with FACS gates providing little information on the respective potencies of individual clones in the sorted pools. We anticipate that the use of de-stabilized FPs containing, for example, C-terminal PEST sequences to accelerate FP turnover, may provide an interesting refinement in this regard as strong, continuously activating CAR clones capable of shifting average steady-state FP accumulation to higher levels may resolve into FACS populations that can be differentiated from clones with more attenuated signalling strengths. Whether such an approach could allow an effective ranking of clones possessing similar characteristics by careful FACS gating remains to be determined.

Beyond the isolation of CAR-active scFvs, consideration should also be given to the optimization potential of all structural elements known to influence the potency and specificity of CAR T cell activation (namely the spacer, TM domain and the specific organisation and sequences of ITAM elements and co-signals). These could, in principle, be diversified and the resultant libraries incorporated into either parallel or sequential (hierarchical/iterative) phenotypic screening cascades, to further tune and optimise CAR properties for any given scFv or scFv panel.

A number of clones emerging from our phenotypic hMSLN CAR discovery screen show diverse context-specific antigen recognition behaviour. Indeed, their individual functional CAR signalling activities do not reliably correlate with either soluble MSLN recognition, or, in the case of clone 13F08, of recombinant bead-immobilized antigen binding. Selective recognition by individual mAbs for GPI-anchored, but not soluble shed or recombinant versions, has been described previously for MSLN and other GPI antigens, suggesting that, at least for this class of membrane proteins, some epitopes are highly conformer sensitive and restricted to the native form^38,43–45^. For antigens such as MSLN that are shed by tumors and accumulate to significant levels in the systemic circulation, CAR T cells constructed from scFv clones that lack appreciable binding to soluble forms should escape potential competition by this ligand ‘sink’, conferring a therapeutic advantage. It therefore follows that, at least for certain membrane-bound protein classes, phenotypic functional screening may represent an attractive approach for identifying CAR-active antibody fragments that are not generally recovered or prioritised by classical in vitro antibody screening technologies, and that may possess favourable selective efficacy and safety characteristics for in vivo and clinical utility.

## Methods

### Cell lines and culture

Unless stated otherwise, all tumor target cell lines were sourced from the ATCC via LGC Standards.

Human endogenous mesothelin (MSLN)-positive target cell lines H-226 (lung carcinoma, MSLN^+^, ATCC® CRL-5826™) and AsPC-1 (ATCC CRL-1682), and MSLN-negative Raji control cells (ATCC CCL-86) were cultured in RPMI-1640 Glutamax (Life Technologies, cat. 61,870,010) containing 10% fetal bovine serum (FBS; Biowest cat. S1810-500) and Pen/Strep (Life Technologies, cat. 15,140,122) at a concentration of 100 U/ml. An AsPC-1 MSLN KO cell pool was generated by CRO (Synthego) by CRISPR/Cas9 targeting of exon 6 using GACACGCUCCGUGCUCAGGC as guide RNA. The KO cell pool was cultured as for wt AsPC-1 cells above. TEM1^+^ A673 (ATCC CRL-1598) and SK-N-AS (ATCC®, CRL-2137) cells were cultured in DMEM Glutamax (Life Technologies, cat. 31,966,047), supplemented with 10% FBS and 100 U/ml Pen/Strep. An A673 line knocked out for TEM1 was generated as for the AsPC-1 KO above by targeting exon 4 using GGAUGAUCCGCACCGCUGUG. Cells were cultured as for the wt A673.

HEK293-6E cells (National Research Council of Canada) were grown in Freestyle F17 medium (Life Technologies, cat. A13835) containing 4 mM GlutaMAX (Life Technologies, cat. 35,050,061), 0.1% Pluronic® F-68 (Life Technologies, cat. 24,040,032) and 25 μg/mL G418 (Fisher Scientific, cat. 10,131,019).

Jurkat NFAT-mCherry (see below) and Jurkat NFAT-Lucia luciferase reporter cells (Invivogen, cat. jktl-nfat) were maintained in RPMI-1640 Glutamax containing 10% fetal bovine serum and 100 U/ml penicillin/streptomycin. All cells were maintained at 37 °C, 5% CO_2_ in a humidified incubator and regularly tested for the absence of mycoplasma (GATC Biotech).

### Generation of a Jurkat NFAT-mCherry phenotypic reporter line

A Jurkat NFAT-mCherry reporter cell line was generated in-house by lentiviral transduction of Jurkat cells (ATCC) with a pRRL-based transgene plasmid containing mCherry under the control of a 6xNFAT inducible promotor, and incorporating a blasticidin gene for the selection of stably transfected cells. Briefly, 293 T human embryonic kidney (HEK) cells were seeded at 1 × 10^6^ in a 6-well tissue culture plate containing 2 ml RPMI supplemented with 10% FCS and allowed to re-attach for 6 h. Transfection of pCMVR8.74 (REV/GAG/POL) and pMD2.G (VSV-G) packaging plasmids (Didier Trono lab, EPFL) in combination with the pRRL transgene plasmid was performed using TransIT-293-Reagent (Mirus, cat. MIR 2704) in Opti-MEM™ I media (Life Technologies, cat. 31,985,062). The resulting virus-containing supernatant was harvested at 48 h post-transfection. For Jurkat cell transduction, 2 ml of filtered viral supernatant was added to 3 ml medium containing 1 × 10^6^ cells in a 25 cm^2^ tissue culture flask in the presence of 10 µg/ml protamine sulfate and incubated overnight at 37 °C. Blasticidin was added to the medium at 5 µg/ml and incubated at 37 °C until the death of equally treated, non-infected Jurkats grown in parallel was observed.

### scFv clones and CAR DNA libraries

Control CAR-mGFPs were constructed using previously reported and characterized scFvs: the human P4 anti-MSLN scFv was extracted from patent US2014301993A1; the anti-CD19 scFv, FMC63, was extracted from patent US7446179A2. An anti-TEM1 scFv clone, 1C1m, is described in patent WO2020243455. Discrete scFvs were cloned into pSTEVe24, a mammalian expression vector harbouring a modular 2nd generation CAR cassette translationally fused to an in-frame monomeric GFP (TagGFP2, Evrogen), and into pSTEVe73, a pRRL-derived lentiviral transduction vector containing the same cassette, using standard methods.

Classical de novo phage-display panning experiments were conducted using a large (2 × 10^10^) naïve scFv human phage display library derived from healthy donor PBMCs and incorporating randomly permutated IgM/IgD VH chains with Vκ and Vλ light chains in the VL-link-VH format (SMD, unpublished). Briefly, Vλ/Vκ chains were amplified using proprietary gene-specific repertoire primers containing NotI (forward) and SalI (reverse) RE sites. Following digestion, these fragments were cloned into pCHV101, a proprietary pUC119-based phagemid vector, containing a p3 M13 phage fusion cassette with an amber (TAG) suppressor codon immediately preceding the p3 ORF and downstream of myc/his epitope tags to allow expression of either scFv (for soluble fragment screening) or scFv-p3 fusions (for phage rescue/display). Stuffer sequences containing RE sites for discrete cloning of VH (via NcoI/AscI) and VL (via NotI/SalI) were separated by a flexible linker enriched in Gly, Ser and Glu residues. Following ligation and electroporation into *E. coli* TG1 (Lucigen, cat. 60,502), the resultant amplified light chain library (~ 10^8^ clones) vector DNA was isolated using Zymopure II Midiprep kit (Zymo Research, cat. D4201) and double-digested with NcoI/AscI. VH chains were amplified with gene-specific primers, similarly digested with NcoI/AscI and cloned alongside the vector-encoded light chains to generate the combinatorial scFv library. Library electroporation, the generation of bacterial glycerol-stocks, and subsequent phage rescue were conducted according to established protocols. Magnetic bead-based panning using Spycatcher-fused antigens has been described previously^46^.

CAR-mGFP libraries were constructed for lentiviral-mediated transfer into Jurkat NFAT-mCherry cells. Briefly, phagemid DNA was isolated from phage display output bacterial glycerol stocks using a ZymoPURE II Midiprep kit (Zymo Research, cat. D4201). The repertoire of scFv ORFs (~ 800 bp) were liberated by NheI/SalI RE digestion for 3 h at 37 °C, purified by agarose gel electrophoresis using a ZymoCLEAN gel DNA recovery kit (Zymo Research, cat. D4002), and ligated into similarly cut pSTEVe73 immediately upstream and in frame with the vector-encoded 2nd-generation CAR-mGFP fused elements. Ligations were performed using T4 DNA ligase (NEB, cat. M0202S) and a 2 h incubation at RT followed by overnight at 16 °C. The ligated products were purified using a Zymo DNA Clean & Concentrator kit (Zymo Research, cat D4006) before either heat shock transformation (45 s at 42 °C) into E.cloni 10G chemically competent cells (Lucigen, cat. 60,107-2), or electroporation into *E. coli* TG1 (Lucigen, cat. 60,502-02). Bulk pSTEVe73 lentiviral vector CAR-mGFP library DNA was isolated from transformed cells using a ZymoPURE Maxiprep kit (Zymo Research, cat. D4202) and concentrated to 1 µg/ml.

### Transfection of HEK293-6E

HEK293-6E cells were transiently transfected with purified pSTEVe24 vectors containing CAR-mGFP constructs using FectoPRO transfection reagent (Polyplus, cat. 116-010) according to the manufacturer’s guidelines. After 48 h post-transfection, CAR expression and recombinant antigen binding were assessed by flow cytometry.

### Transduction of Jurkat lines and primary human T cells

Lentivirus particles containing CAR-mGFP constructs and libraries were produced in HEK293T cells transiently transfected with either individual CAR-mGFPs or library pools housed in pSTEVe73 together with pCMVR8.74 and pMD2.G packaging plasmids (Didier Trono lab, EPFL) using Turbofect reagent (Life Technologies, cat. R0532) according to the manufacturer’s guidelines. Viral particles were harvested after 48 h and concentrated by ultracentrifugation. Transduction of Jurkat NFAT-Lucia and Jurkat NFAT-mCherry was performed using either concentrated lentiviral supernatants (single CAR-mGFP clones) or, for CAR library construction, lentivirus supernatants were diluted in a twofold dilution series and added immediately to 1 × 10^6^ Jurkat NFAT-mCherry cells. Transduced cells were expanded and assessed for GFP expression by flow cytometry after 7 days. Cell library populations containing 10–20% transduced cells were selected and expanded for another 3–7 days before being used for phenotypic screening (see below).

Primary human T cells were isolated from fresh buffy coats obtained from healthy volunteers via the Inter-regional Blood Transfusion SRC (Lausanne) facility. Peripheral blood mono-nucleated cells (PBMCs) were isolated by density centrifugation using Lymphoprep (Axonlab). T cells were subsequently extracted by magnetic separation using a human pan-T cell isolation kit (Miltenyi Biotec, cat. 130-096-535). Prior to transduction, 0.5 × 10^6^ of freshly isolated CD3^+^ T-cells were plated in tissue culture-treated 48-well plates and expanded with CD3/CD28 dynabeads (bead:cell ratio of 2:1; Life Technologies, cat. 11161D). Beads were removed at day 5 and CAR-mGFP lentiviral supernatants were used to transduce the CD3/CD28-bead activated T-cells which were then maintained in complete media supplemented with human recombinant human IL2 (50 IU/mL, Glaxo IMB) for a further 3 days. The expanded CAR-mGFP T-cells were supplemented with complete media containing 10 ng/ml of IL-7 and IL-15 (Miltenyi Biotec, cat. 130-095-367 and 130-095-765) and incubated for 1–2 weeks at 37 °C, 5% CO_2_ prior to performing killing assays directly (without cryopreservation). Cells were split and fed every 2–3 days with fresh media plus IL-7/IL-15. Transfection efficiencies were determined based on the proportion of GFP^+^ cells.

### Jurkat NFAT-mCherry phenotypic screening

Jurkat NFAT-mcherry CAR-mGFP cell libraries were first ‘cleaned’ by stimulating with irrelevant, antigen-negative HEK293-6E cells followed by sorting to enrich for the non-activated GFP^+^/mCherry^-^ population (‘NS’, negative sort). Typically, 2–3 × 10^6^ HEK293-6E cells were co-cultured with an equivalent number of CAR-mGFP library Jurkat cells in sterile 6-well plates in a total volume of 2 ml. Additionally, non-transduced Jurkat NFAT-mCherry cells were maximally activated with phorbol myristate acetate (PMA)/iomomycin cell stimulation cocktail (Thermo Fisher Scientific, cat. 00-4970-03) as a positive control. After 24 h incubation at 37 °C, 5% CO_2_, the cells were resuspended and washed once with 2 ml FACS buffer (5% FBS in PBS) before proceeding to FACS sorting of GFP^+^/mCherry^-^ cells into single tubes. The resulting cleaned Jurkat libraries were expanded for 7 days and then stimulated with target cells expressing the antigen of interest (‘CS’, positive cell sort). Briefly, adherent target cells (A673 cells for CAR-mGFP Jurkat libraries targeting the hTEM1 mucin stalk, TEM1(Δn) and H-226 or AsPC-1 cells for Jurkat libraries targeting hMSLN) were harvested with trypsin–EDTA, counted and typically adjusted to 2–4 × 10^6^ cells in a 6-well plate. Library-expressing Jurkat populations were added at 2–4 × 10^6^ cells to obtain an effector-to-target (E:T) ratio of 1:1 with stimulation allowed to proceed for 24 h at 37 °C, 5% CO_2_ before harvesting, washing and FACS sorting of cells as described above. Again, non-transduced Jurkat NFAT-mCherry cells stimulated with PMA/ionomycin served as a positive activation control. GFP^+^/mCherry^+^ cells from each library were sorted and expanded for 14 days prior to one or more additional cycles of positive cell stimulation and sorting. For all sorting experiments, dead cells were excluded by staining with 4′,6-Diamidino-2-phenylindole (DAPI, 1:2000 dilution). All cell sorting was performed using a FACSAria III cell sorter equipped with FACSDIVA software (BD Biosciences).

### Retrieval and cloning of phenotypically enriched CAR scFvs

Following phenotypic screening and cell sorting, mRNA was isolated from 10^6^–10^7^ bulk Jurkat library cells using the Dynabeads mRNA DIRECT purification kit (Thermo Fisher Scientific, cat. 61,011) according to the manufacturer’s instructions. Subsequently, 1 µg of the resulting mRNA served as the template for cDNA synthesis using the PrimeScript 1st strand cDNA kit (Takara, cat. 6110B) and oligo-dT primers. The scFv sequences were amplified from the cDNA template using Phusion High-Fidelity PCR Master Mix with GC Buffer (NEB, cat. M0532S) and appropriate flanking primers situated upstream in the scFv signal peptide (Forward) and downstream in the CD28 extracellular spacer region (Reverse). Successfully amplified scFv pools were purified from 1% agarose gels using a ZymoCLEAN gel DNA recovery kit (Zymo Research, cat. D4002) and cloned into the pCHV101 phagemid vector using NcoI/SalI. *E. coli* TG1 cells were electroporated, and discrete ampicillin-resistant colonies were picked for high-throughput plate sequencing (Microsynth).

### Production of soluble scFvs

Single colonies of *E. coli* TG1 cells (Lucigen, cat. 60,502) containing phagemid vectors expressing scFvs under control of a Lac promotor were picked by robot (Qpix420, Molecular Devices) into 96-well PP U-form microplates (Greiner, cat. 650,201) containing 200 µL of Terrific Broth (TB), 2% glucose, 100 μg/ml ampicillin and cultures were grown overnight at 30 °C with shaking at 750 rpm (70% humidity). Turbid overnight cultures were used to inoculate 170 μl TB containing 0.1% glucose and 100 μg/ml ampicillin using QRep 96 Pin Replicators (Molecular Devices, cat. X5051). The cultures were grown in 96-well PP U-form microplates with shaking at 30 °C for 6 h until the OD_600_ reached 0.6. The expression of scFvs was induced by adding 50 μl per well of 440 µM Isopropyl-β-D-thiogalactopyranoside (IPTG; Merck, cat. 420,322) to achieve a final concentration of ~ 100 μM. Expression was allowed to continue for 16–18 h at 30 °C with shaking at 750 rpm, 70% humidity before centrifuging plates to allow the harvesting of clarified media for subsequent binding assay.

### Screening of soluble scFvs for target cell binding

Adherent target cells were detached using 10 mM EDTA and 0.1 × 10^6^ pre-blocked cells were resuspended in 100 µl scFv-containing *E. coli* TG1 expression supernatants diluted 1:2 in assay buffer. After 30 min of incubation on ice, the wells were washed 1 × with FACS buffer (PBS/5% FBS/0.02% sodium azide). Alexa Fluor 647-conjugated anti-his tag antibody (100 µl, 1:2500; Genscript, cat. A01802-100) was added for 30 min on ice to detect binding of his-tagged scFvs. The samples were washed 2 × with FACS buffer and data was acquired immediately on an Intellicyt iQue TM Screener PLUS instrument (5 s sampling; 1 µl/s) with dead cells excluded by DAPI staining (1:2000).

### Antigen binding assay (FACS)

Cells were collected and washed once with FACS buffer (PBS/5% FBS /0.02% sodium azide). Tagged recombinant target antigens (bio-hTEM1 (Fierle et al. 2019), bio-hCD19 [AAs 20–291; Acro Biosystems, cat. CD9-H82E9], bio-hMSLN [AAs 296-580; Acro Biosystems, cat. MSN-H82E9], hMSLN-his [AAs 1-286; SinoBiologics, cat. 13,128-H08H-50], hMSLN [AAs 296-580; R&D Systems, cat. 3265-MS-050]) were added to the cells at 1 µg/ml in FACS buffer. After 30 min of incubation on ice followed by three wash steps with 100 µl FACS buffer, antigen binding was revealed using either APC-conjugated streptavidin (1:2000; Biolegend cat. 405,207) or anti-Histag-Alexa647 (1/1000; Genscript, cat. A01802-100), diluted in FACS buffer. Stained cells were incubated for 30 min on ice and washed a further three times. Immediately before data acquisition, dead cells were stained with 4′,6-Diamidino-2-phenylindole (DAPI, 1:2000 dilution). Data was acquired using an LSR-II flow cytometer equipped with FACSDIVA software (BD Biosciences). Data analysis and plotting were carried out using FlowJo v10 (FlowJo LLC).

### NFAT activation assay

For co-culture NFAT activation assays, adherent cells were detached using 10 mM EDTA, counted and re-suspended in fresh, complete culture medium. Target cells (0.5 × 10^6^) were seeded into 24-well assay plates and 10^6^ CAR-transduced Jurkat NFAT-mCherry cells were added in a total volume of 1 ml complete RPMI. After 24 h of co-culture, the cells were harvested and washed once with 2 ml FACS buffer (5% FBS in PBS). Immediately before data acquisition on an LSR-II flow cytometer (BD Biosciences), dead cells were stained with DAPI (1:2000). Alternatively, the functional activation potential of CAR constructs was assessed using Jurkat NFAT-Lucia luciferase reporter cells (Invivogen, cat. jktl-nfat), as described previously^46^.

### Primary CAR T cell end-point cytotoxicity and activation assays (Antares reporter)

For FACS-based assays, effector anti-MSLN CAR-mGFP T cells were co-incubated overnight in flat 96-well plates at a 5:1 ratio with a H-226 target cell line transduced for constitutive expression of a CyOFP1-Nanoluc fusion protein (Antares)^47^. Cells were recovered with 10 mM EDTA and transferred into V-bottomed 96-well plates (Corning, cat. 3894) and washed once with PBS before being blocked with 100 μl of FACS buffer (5% FBS in PBS/5 mM EDTA) for 30 min on ice. Cells were washed once with FACS buffer and then stained with human anti-hCD25-PE (1:1000; Biolegend, cat. 302,606) for 30 min on ice. Stained cells were washed once in FACS buffer and re-suspended in 30 μl of DAPI (4′,6-diamidino-2-phenylindole, 1:2000 dilution), just prior to data acquisition. Living target H-226 cell numbers and CD25-positivity of effector CAR T cells were resolved by FSC/SSC, and by the expression of the fluorescent marker CyOFP1. Data acquisition was performed using an iQue Screener Plus (Sartorius, IntelliCyt) with plate agitation (1800 rpm every 3rd well) and a 6 s sample sipping time. Surviving target cells were quantified as the absolute number of DAPI-negative events while the efficiency of anti-MSLN CAR-mGFP T cell transduction was measured as the % of T cells expressing GFP.

For assessment of killing by Nanoluc luciferase release, Antares-transduced H-226 target or MSLN^−^ Raji control cell lines were co-cultured with anti-MSLN CAR-mGFP T cells for 24 h at an E:T ratio of 5:1. Subsequently, supernatants were transferred to white plates containing an equal volume of the Nanoluc substrate, furimazine (Promega, cat. N1110). Luminescence was read in endpoint mode for 10 s using a BioTek synergy H4 hybrid microplate reader. Maximum potential killing response was estimated by lysis of Antares cell lines using 0.8% Triton-X-100 (lysis buffer) in the absence of CAR T cells.

Induced IFN-γ secretion was quantified by sandwich ELISA (Biolegend, cat. 430,102) using 24 h supernatants from effector anti-MSLN CAR-mGFP CAR T cells and H-226 target tumor cell co-cultures. The assay was performed according to the manufacturer’s protocol. End-point absorbance data were acquired on a BioTek H1MFG Synergy plate reader using TMB as a substrate for the HRP-conjugated secondary antibody.

## Supplementary Information

Supplementary Information.

## Data Availability

All data generated or analyzed during this study are included in this published article.
