# Supplementary material for: A cell-based phenotypic library selection and screening approach for the de novo discovery of novel functional chimeric antigen receptors
Source: Sci Rep. 2022 Jan 21;12:1136. doi: 10.1038/s41598-022-05058-5 (PMC8782825; doi:10.1038/s41598-022-05058-5)
Supplement: Supplementary file 1 — Supplementary Information. [file 41598_2022_5058_MOESM1_ESM.pdf]

SUPPLEMENTARY INFORMATION [FIGURES S1-S6; TABLE S1; METHODS]

A cell-based phenotypic library selection and screening approach for the *de novo* discovery of novel functional chimeric antigen receptors

Julie K. Fierle[1*], Johan Abram-Saliba[1*], Vasileios Atsaves[1], Matteo Brioschi[1], Mariastella deTiani[1], Patrick Reichenbach[2], Melita Irving[2], George Coukos[3,4] and Steven M. Dunn[1,5]
* These authors contributed equally to this work

Author affiliations
[1] LAbCore Immunoglobulin Discovery Platform, Department of Oncology, Ludwig Institute for Cancer Research Lausanne, University of Lausanne, 1066 Epalinges, Switzerland.
[2] Department of Oncology, Ludwig Institute for Cancer Research Lausanne, University of Lausanne, 1066 Epalinges, Switzerland.
[3] Department of Oncology, Ludwig Institute for Cancer Research Lausanne, Lausanne University Hospital and University of Lausanne, 1005 Lausanne, Switzerland.
[4] Department of Oncology, Centre Hospitalier Universitaire Vaudois (CHUV), 1011 Lausanne, Switzerland.
[5] Department of Oncology, Ludwig Institute for Cancer Research Lausanne, Lausanne University Hospital and University of Lausanne, 1066 Epalinges, Switzerland.

Correspondence and requests for materials should be addressed to S.M.D. (steven.dunn@chuv.ch)

Supplementary Figure S1

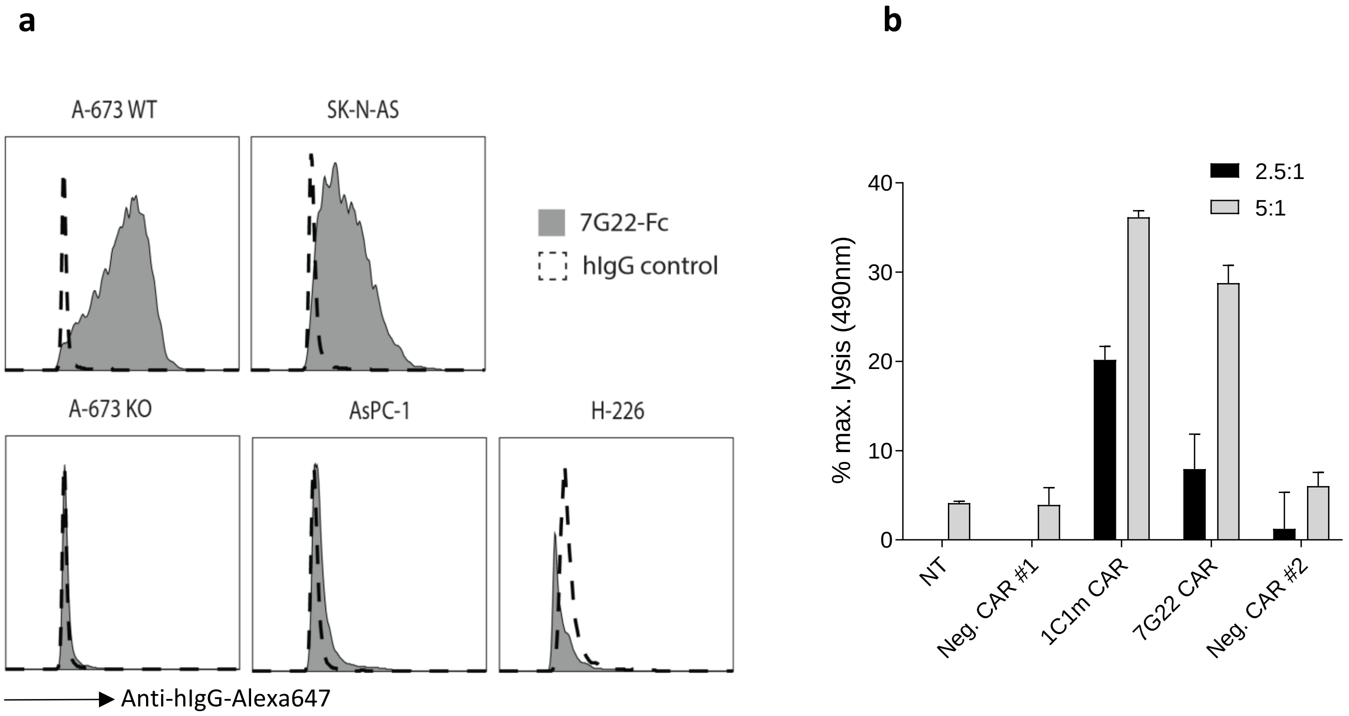

(a) FACS staining for TEM1 expression on cognate TEM1⁺ (A673, SK-N-AS) and TEM1-negative control cells (A673 KO, AsPC-1, H-226) using 7G22 scFv-Fc fusion (1 μg/ml). (b) Primary T cell-mediated killing assay of TEM1⁺ A673 cells by the phenotypically rescued CAR-mGFP clone 7G22 at two E:T ratios. The degree of killing is measured by lactate dehydrogenase (LDH) release and a colorimetric substrate. Neg. CAR#1/2, irrelevant scFv CARs; 1C1m CAR, positive anti-TEM1 control. Data are represented as mean ± SD from triplicate discrete assay points.

Supplementary Figure S2

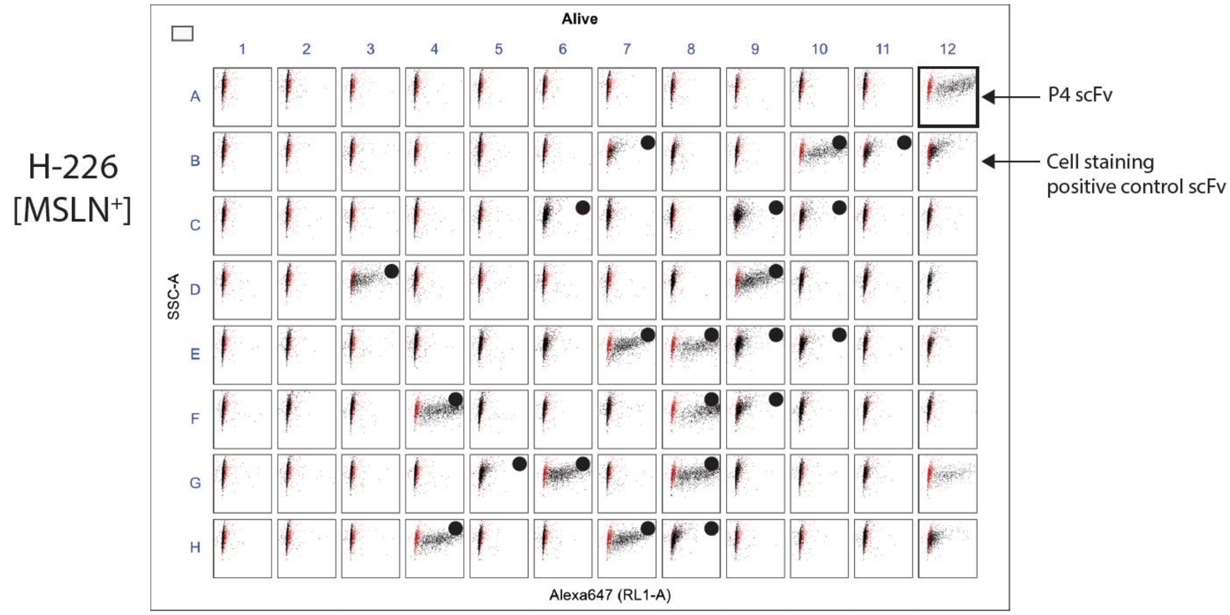

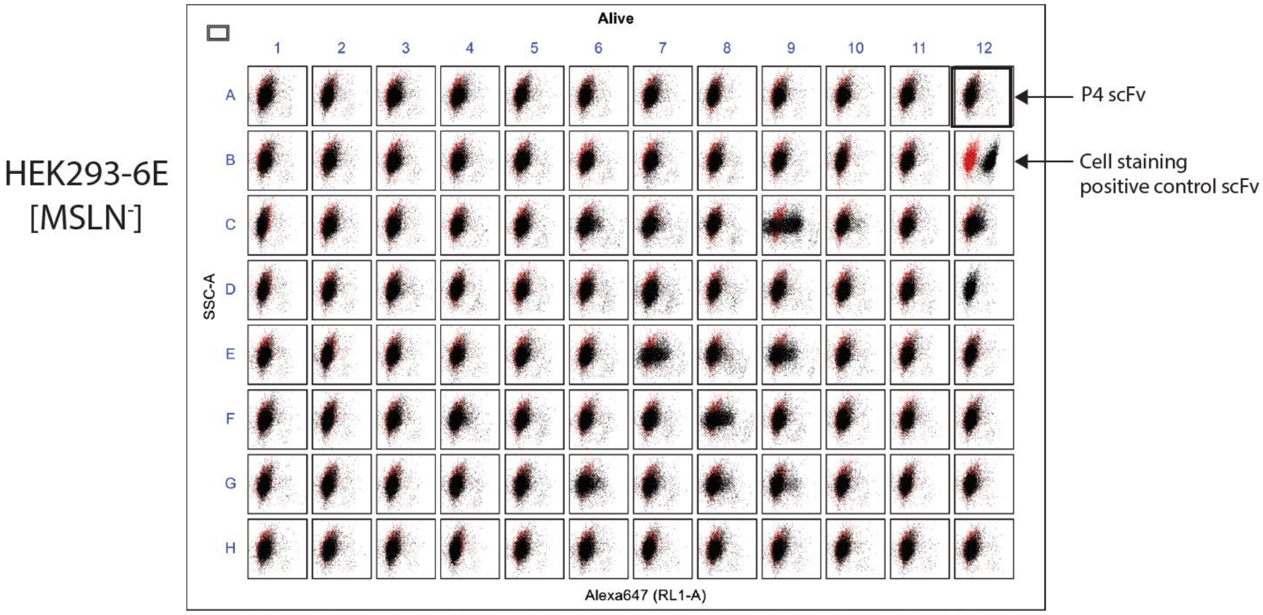

Illustrative iQue flow cytometry screening of recovered scFvs from anti-MSLN CAR phenotypic sorting. Bacterially expressed soluble scFv clone supernatants are screened in duplicate for binding to cognate target (H-226, upper) and negative control (HEK293-6E, lower) cells. For each well, cells in the presence of a scFv clone (black) are superimposed on a control cell population in the absence of scFv (red). Appreciable binding is indicated by a right-shifted black cell population. Bound scFvs are stained using

anti-Histag-Alexa647. Clones of interest binding H-226 but not HEK293 are indicated by closed filled circles. P4 scFv (well A12) is a benchmark anti-MSLN scFv. Alive cells defined as (FCS/SSC) -> singlets (FCS-A/FCS-H) -> singlets (SSC-A/SSC-H) -> DAPI-negative.

Supplementary Figure S3

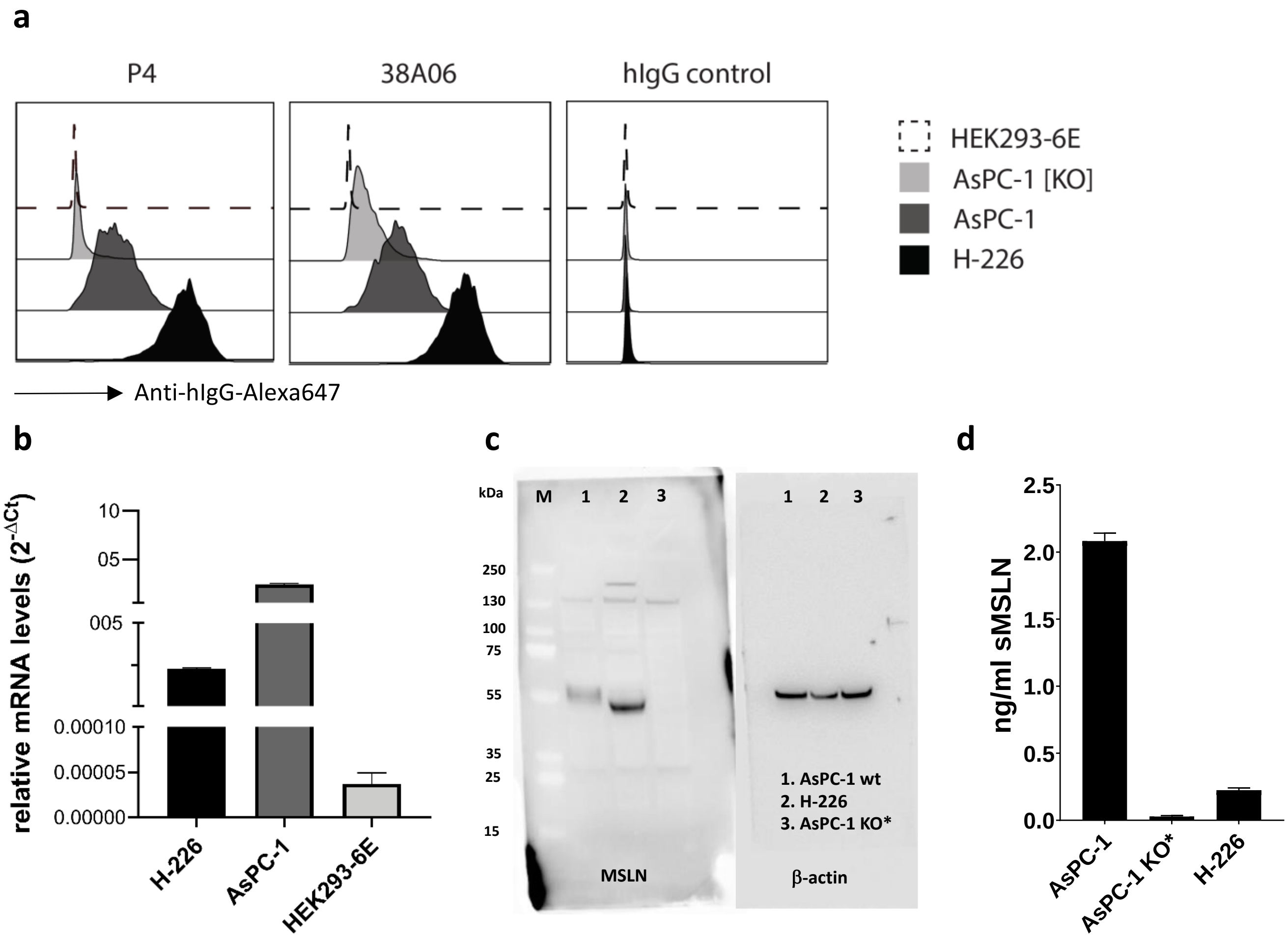

(a) FACS staining for shed (s)MSLN expression on cognate and control cells using scFv-Fc fusions. Clone P4 is described in the main text; clone 38A06 was generated in-house via a classical phage display approach; purified scFv-Fcs were used at 0.5 μg/ml. (b,c) Comparative expression of MSLN in AsPC-1 and H-226 cells. (b) RT-PCR quantitation of relative mRNA levels. (c) Western blot performed on solubilized whole cell extracts. (d) Quantitation of soluble MSLN detected in 60 h clarified cell media by sandwich ELISA. AsPC-1 KO* is a line sorted from the parental AsPC-1 polyclonal MSLN CRISPR KO pool described

in the main text. Unlike the AsPC-1 KO pool, AsPC-1 KO* shows no residual staining with MSLN antibodies

Supplementary Figure S4

a

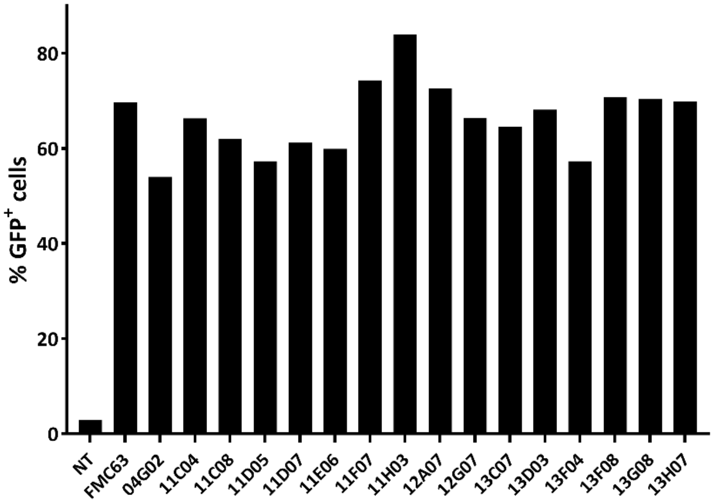

b

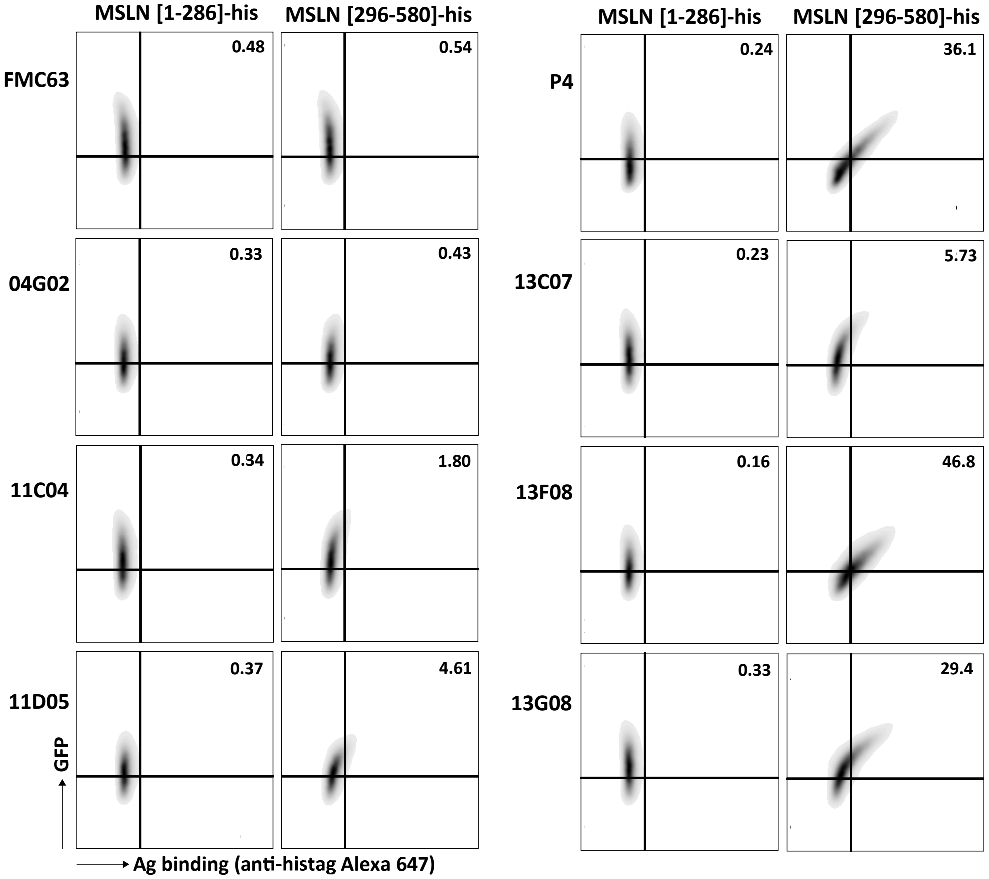

(a) Relative expression of discrete anti-MSLN CAR-mGFP clones in transduced Jurkat NFAT-mCherry cells as measured by mature GFP levels. (b) Binding of recombinant purified soluble MSLN fragments to Jurkat cells expressing discrete anti-MSLN CAR-mGFP clones. MSLN[1-286], negative control fragment comprising the cleaved megakaryocyte-potentiating factor; MSLN[296-580], target fragment comprising the mature mesothelin extracellular antigen.

Supplementary Figure S5

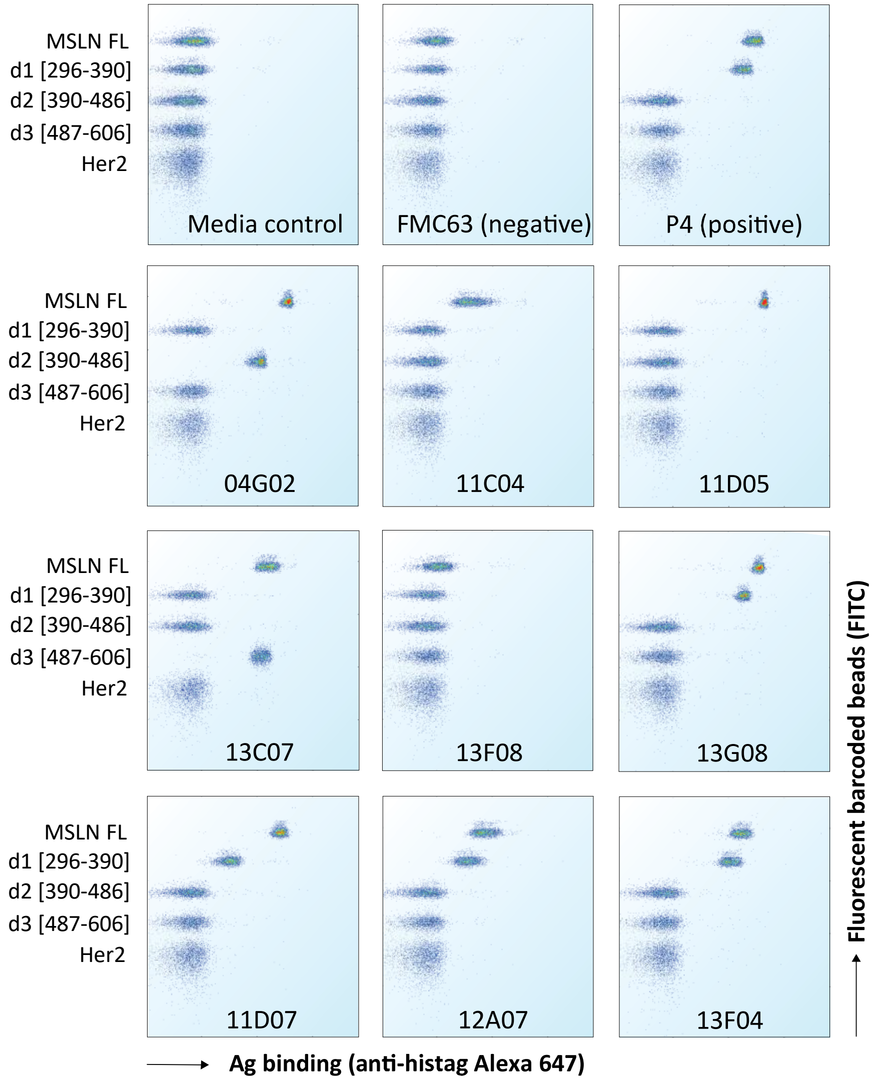

Epitope profiling by bead-based multiplex iQue FACS screening assay of phenotypically-selected CAR-active scFvs towards full length mature mesothelin and discrete isolated ECD domains.

Supplementary Figure S6

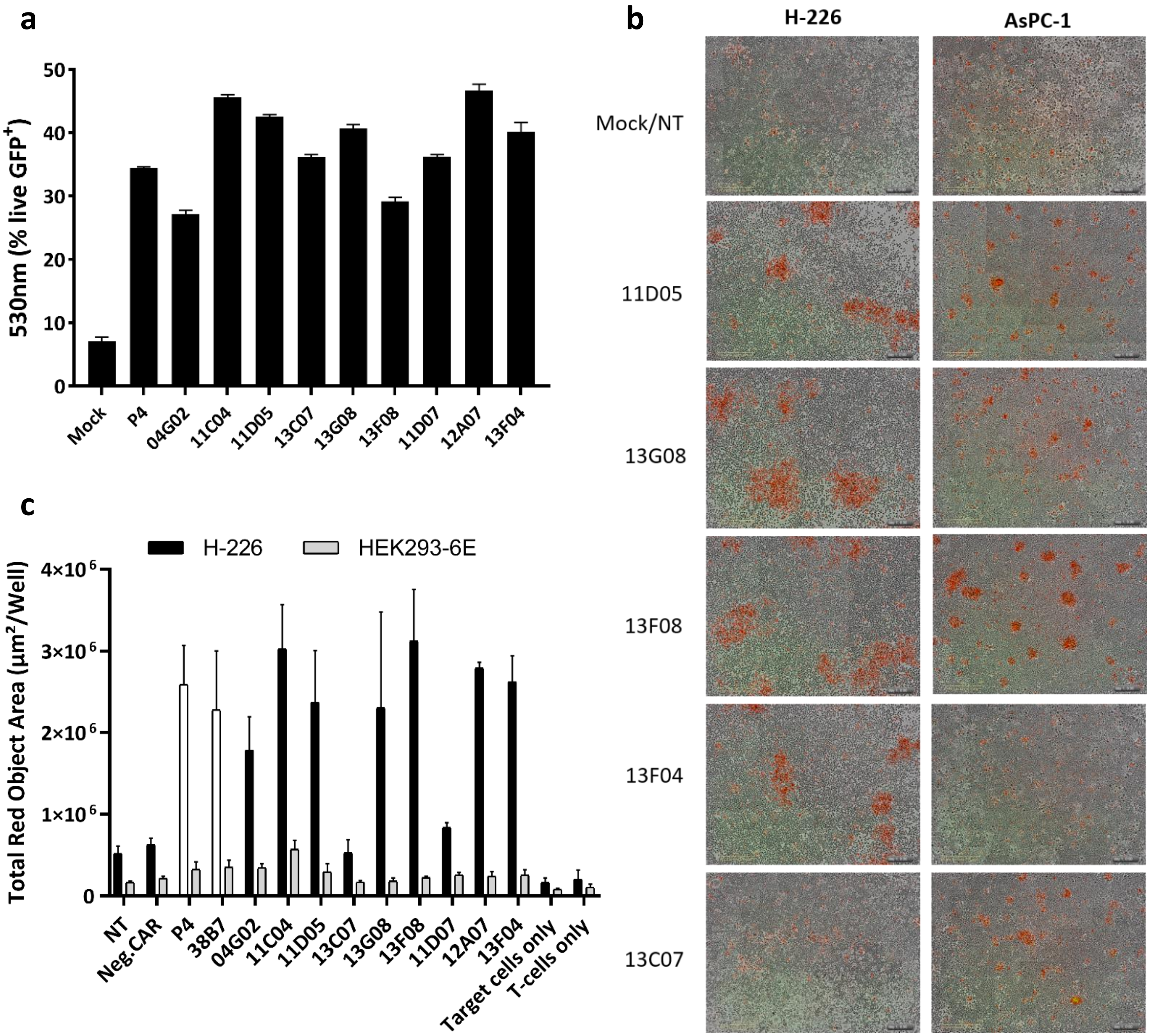

(a) Relative expression of discrete anti-MSLN CAR-mGFP clones in transduced primary human pan T cells as measured by mature GFP levels. (b,c) Killing of MSLN$^+$ target cells by phenotypically-selected CAR-mGFP clones as visualized by Incucyte assay. Red clusters indicate killing foci after 24h with quantification shown in (c) for H226 in comparison to MSLN-negative HEK293-6E cells. P4 and 38B7 are included as representative clones isolated by classical phage display screening procedures. Data are represented as mean ± SD from triplicate discrete assay points.

Supplementary Table S1

Method	Clone ID	K_{on} (1/Ms)	K_{off} (1/s)	KD (nM)
Phage Display	*P4 (benchmark)*	*1,68E+05*	*6,12E-04*	*4*
Phage Display	38B07	4,27E+04	5,66E-04	10
Phage Display	38A06	2,91E+05	1,38E-03	5
CAR phenotypic	04G02	3,42E+05	4,66E-03	10
CAR phenotypic	11C04	7,41E+04	3,12E-03	40
CAR phenotypic	11D05	1,74E+05	4,81E-03	30
CAR phenotypic	13C07	4,12E+05	2,22E-03	5
CAR phenotypic	13G08	2,83E+05	2,55E-03	9
CAR phenotypic	13F08	NA	NA	No binding
CAR phenotypic	11D07	6,32E+04	1,62E-03	30
CAR phenotypic	12A07	1,13E+05	1,46E-03	10

Monovalent kinetic parameters determined by biolayer interferometry for monovalent anti-MSLN scFvs. Clones in shaded rows originate from classical phage display selection and screening performed on recombinant immobilized MSLN. Clone P4 is described in the main text; clones 38A06 and 38B07 were generated in-house using a classical phage display procedure. Clones labelled as 'CAR phenotypic' originate from this work.

METHODS

MSLN multiplex domain scFv binding assay

Discrete scFv clones were characterized for hMSLN domain binding by a mix-and-read multiplex bead assay. Briefly, hMSLN domain regions were fused to SpyCatcher to generate full length MSLN (AA 296-606), region 1 (AA 296-390), region 2 (AA 391-486), and region 3 (AA 487-606) recombinant antigens. As an irrelevant control, a SpyCatcher fusion was generated to a fragment of hHer2 (domains 3 and 4). Fusion proteins were expressed from HEK293-6E cells and covalently captured directly from supernatants on distinct fluorescently barcoded streptavidin-coated beads (Spherotech, cat. SVFB-2552-6K) using a direct capture and immobilization (dCI) protocol described previously[46]. The antigen-coated bead populations were combined and blocked in assay buffer (PBS/2% BSA/0.1% Tween-20/2mM EDTA) for 30 min at RT. Multiplexed beads (10 μl) were added to wells of a 96-well plate, followed by an equal volume of scFv-containing *E. coli* TG1 expression supernatants diluted 1:16 in assay buffer. After incubating for 30 min at RT, bound scFvs were detected by addition of 10 μl of an iFluor647-conjugated anti-his tag antibody (GenScript, cat. A01802-100) at a final dilution of 1:2500 in assay buffer. Following a further 30 min incubation at RT in the dark, the sample plate was read directly on an Intellicyt iQue TM Screener PLUS instrument (5 s sampling; 1 μl/s). Bead populations displaying different immobilized SpyC-antigens were resolved by their respective barcoding fluorescence intensity (FITC), and scFv binding to each population was assessed by iFluor647-mediated median fluorescence intensity (MFI). Data analysis was performed using Forecyt software (Intellicyt).

MSLN qRT-PCR and Western Blot analysis

For qRT-PCR analysis, mRNA was prepared from 10^7 lysed and homogenized cells using a Qiagen RNeasy Mini kit (cat. 74106) according to the manufacturer's instructions. Reverse transcription to cDNA was performed using a Takara Primescript First Strand cDNA synthesis kit (cat. 6110B) according to the manufacturer's instructions. Briefly, 5 μM oligo dT primer was annealed to 1 μg total mRNA in a total volume of 10 μl containing 1mM each dNTP, by incubating at 65 °C for 5 min. 20 units RNase inhibitor and 200 units PrimeScript reverse transcriptase were added to the annealed RNA/primer mixture and the reaction was allowed to take place for 1 h at 42 °C. The reaction was stopped by heating to 70 °C for 15 min and the samples were immediately cooled on ice. For TaqMan qPCR, approximately 50 ng cDNA were mixed with 10 μl TaqMan Fast Universal Master Mix (Applied Biosystems, cat. 4352042) and 1 μl

FAM-MGB-TaqMan probe in a total reaction volume of 20 µl. Specific detection probes were obtained from ThermoFisher Scientific (hMSLN: Hs00245879_m1, cat. 4453320; hGAPDH: Hs02786624_g1, cat. 4331182). Real-time PCR amplification of hMSLN and the hGAPDH housekeeping gene was performed in a 7500 Fast RT-PCR machine (Applied Biosystems) and relative gene expression levels in different cell lines were analyzed by comparative threshold cycle (Ct) quantification. Expression levels are presented relative to GAPDH.

For the detection of cell MSLN protein, whole cell extracts from $3x10^6$ cells were prepared using RIPA Lysis and Extraction Buffer (ThermoFisher Scientific, cat. 89900) according to the manufacturer's instructions. The concentration of the isolated proteins was determined using a BCA Protein Assay Kit (ThermoFisher Scientific, cat. 23225). Proteins were separated by SDS-PAGE on a 4-12% BisTris NuPAGE gel (Life Technologies, cat. NP0321BOX) and subsequently transferred to a polyvinylidene difluoride (PVDF) membrane using an iBlot™ 2 Gel Transfer Device (ThermoFisher Scientific, cat. IB21001). The membrane was blocked (PBS, 0.1% Tween-20, 5% BSA) for 1 h before being challenged for 1 h with the anti-MSLN monoclonal antibody clone MN-1 (Merck Millipore, cat. MABC1140; 1:1,000). In parallel, staining of identically loaded lanes on the same blot was conducted using anti-β-actin (Santa Cruz Biotechnology, cat. sc-47778; 1:2,500) as a loading control. Following the primary antibody incubations, the signals were developed using an anti-mouse-HRP secondary conjugate (Sigma-Aldrich, cat. A9917-1ML; 1:10,000) and SuperSignal™ West Pico PLUS Chemiluminescent Substrate (ThermoFisher Scientific, cat. 34580). Visualization was performed on a Fusion FX imaging system (Vilber).

Detection of shed MSLN by Sandwich ELISA

A commercially available kit (ThermoFisher Scientific, cat. EH322RB) was used to detect the level of human mesothelin in cell culture supernatants according to the manufacturer's instructions.

Briefly, clarified media from 60 h-grown cultures (seeded with $1x10^6$ cells) was diluted 1:5 in fresh media and 100 µL were added to the appropriate capture wells of the kit plate. The plate was incubated at 4°C overnight with gentle shaking. The following day the solution was discarded and the wells washed 4x with kit wash buffer. After the washing step 100 µL of biotin conjugated sandwich reagent was diluted 80-fold into each well and incubated the plate for 1 hour at room temperature with gentle shaking before repeating the above was step. Well signals were developed using the kit Streptavidin-HRP solution (100 µl/well) with incubation for 45 min at room temperature with gentle shaking. Following washing, colour was developed using TMB substrate with incubation in the dark for 8 minutes followed by the addition of stop solution. Absorbance was read at 450nm on a Synergy H1 (Biotek) plate reader.

Shed MSLN was determined by interpolation from a standard curve generated from the provided kit reagents.

FACS staining of cells using purified scFv-Fc fusions

Adherent target cells were detached using 10 mM EDTA and blocked with FACS buffer (PBS/5% FBS) for 30 min of incubation on ice. 200 000 cells were distributed per well, span down and resuspended in 100 µl of either 1 µg/mL 7G22-Fc (for TEM1) or 0.5 µg/ml P4-Fc and 38A06-Fc (for MSLN). hIgG (1 µg/ml; Abcam, Cat. ab98981) was used for control stainings. After 45 min of incubation on ice followed by two wash steps with 100 µl FACS buffer, anti-hIgG Fc-Alexa647 (1:200; Jackson Immunoresearch, Cat. 109-605-098) diluted in FACS buffer was added to the cells. Stained cells were incubated for 45 min on ice and washed a further two times. Immediately before data acquisition, dead cells were stained with 4',6-Diamidino-2-phenylindole (DAPI, 1:2000 dilution). Data was acquired using an LSR-II flow cytometer equipped with FACSDIVA software (BD Biosciences). Data analysis and plotting were carried out using FlowJo v10 (FlowJo LLC).

Primary CAR T cell cytotoxicity (LDA release assay)

Adherent target cells ($2x10^4$) were seeded in 96-well flat-bottom plate and allowed to attach for ~20 h. When approximately 30 % confluency was observed, $6x10^5$ or $1.25x10^6$ purified and expanded CAR-mGFP T cells were added to the plate to reach an E:T ratio of 2.5:1, or 5:1, respectively. After 24 h of co-culture, specific target cell killing was assessed by measuring LDH release with the CytoTox 96 kit (Promega, cat. G1780), following the manufacturer's instructions. Briefly, 50 µl clarified co-culture supernatant was mixed with 50 µl CytoTox 96 Reagent and incubated at RT for 30 min in the dark. The reaction was stopped by adding 50 µl of Stop solution and LDH activity was quantified at 490 nm on a BioTeK H1MFG Synergy plate reader. Maximum lysis was determined by the addition of 10% Triton X-100 to control wells. Background signal was subtracted from all samples and cell killing (corrected for spontaneous release by target and effector cell control wells) was calculated as a percentage of maximum lysis.

Visualization of target cell killing

$MSLN^+$ target cell killing was visualized using cell imaging (Incucyte system, Essen Bioscience). H-226 or AsPC-1 target cells (100 µl/well) were seeded in 96-well plates at a density of $0.15x10^6$ cells/ml in 100 µl complete medium. The following day, cell density had reached $0.2x10^6$ cells/well. Supernatant was

removed and 50 µl of CytotoxRED dead-cell staining reagent (500 nM; Essen Bioscience, cat. 4632) was added to each well together with 50 µl of effector anti-MSLN CAR-mGFP CAR T cells ($1x10^6$ cells/ml) in complete medium resulting in a 5:1 E:T ratio. Plates were returned to the incubator for 30 min to allow the combined cells and dye to equilibrate, before being transferred to the Incucyte system for 3 days. Cell death was monitored as an increase in red fluorescence with time and analyzed with the Incucyte integrated analysis software (Incucyte, ZOOM2016A). Kill slopes were determined using the Total Red Image Integrated Intensity per Image data. Representative micrographs were taken after 24h of incubation.

Determination of binding kinetics

Affinity measurement for anti-MSLN interactions were performed using biolayer interferometry analysis on a GatorPrime instrument (Gator Bio, Inc.). Briefly, hMSLN-SpyCatcher was complexed with biotinylated SpyTag in solution, buffer exchanged to remove free bio-SpyTag, and immobilized onto Streptavidin Probes (Gator Bio, Cat. 160002) at a density of 0.6 RU. The binding of monovalent anti-MSLN scFvs (fused to an irrelevant carrier scFv in a BiTE© arrangement) was conducted over several concentrations (typically 0 nM, 12.5 nM, 25 nM, 50 nM) in PBS/0.1% Tween-20, with association for 180 s (1000 rpm) and dissociation for 120 s (1000 rpm). Probes were regenerated between experiments using 3 cycles of 10 mM glycine-HCl, pH 1.5 (5 s, 1000 rpm).
